# Páramo *Calamagrostis* s.l. (Poaceae): An updated list and key to the species known or likely to occur in páramos of NW South America and southern Central America including two new species, one new variety and five new records for Colombia

**DOI:** 10.3897/phytokeys.122.33032

**Published:** 2019-05-28

**Authors:** Steven P. Sylvester, Robert J. Soreng, William J. Bravo-Pedraza, Lia E. Cuta-Alarcon, Diego Giraldo-Cañas, Jose Aguilar-Cano, Paul M. Peterson

**Affiliations:** 1 College of Biology and the Environment, Nanjing Forestry University, Long Pan Road No. 159, Nanjing, 210037, China Nanjing Forestry University Nanjing China; 2 Royal Botanic Gardens, Kew, Richmond, Surrey, TW9 3AE, UK Royal Botanic Gardens Kew United Kingdom; 3 Department of Botany, National Museum of Natural History, Smithsonian Institution, Washington DC 20560, USA National Museum of Natural History, Smithsonian Institution Washington United States of America; 4 Grupo Sistemática Biológica, Herbario UPTC, Escuela de Biología, Facultad de Ciencias, Universidad Pedagógica y Tecnológica de Colombia, Avenida Central del Norte 39-115, Tunja-Boyacá, Colombia Universidad Pedagógica y Tecnológica de Colombia Tunja Colombia; 5 Instituto de Ciencias Naturales, Universidad Nacional de Colombia, Apartado 7495, Bogotá D. C., Colombia Universidad Nacional de Colombia Bogotá Colombia; 6 Colecciones Biológicas, Instituto de Investigación de Recursos Biológicos Alexander von Humboldt, Claustro de San Agustín, Carrera 8 No. 15-08, Villa de Leyva, Boyacá, Colombia Instituto de Investigación de Recursos Biológicos Alexander von Humboldt Villa de Leyva Colombia

**Keywords:** Andes, checklist, Costa Rica, *
Deschampsia
*, *
Deyeuxia
*, Ecuador, Gramineae, grassland, Panama, pastizal, Peru, taxonomia, taxonomy, Venezuela

## Abstract

*Calamagrostis* (syn. *Deyeuxia*), as traditionally circumscribed, is one of the most speciose genera from páramo grasslands of northwest South America and southern Central America and often dominates these high-elevation habitats. However, it remains difficult for researchers to accurately identify the species due to a lack of floristic treatments for most of the countries containing páramo, with the distribution of many species still very poorly known. In an effort to ameliorate this, we present an updated list and identification keys in English and Spanish (as electronic appendix) to the species of *Calamagrostis* s.l. known or likely to occur in the páramos of Peru, Ecuador, Colombia, Venezuela, Costa Rica and Panama. Fifty-four species are accepted, constituting 47 species currently circumscribed in *Calamagrostis* and seven species recently transferred to *Deschampsia*. Included within this are two new species, *Calamagrostiscrispifolius* and *Deschampsiasantamartensis*, which are described and illustrated. Both new species are found in páramos of the Sierra Nevada de Santa Marta (departamento Magdalena), on the northernmost tip of Colombia, with *C.crispifolius* also found in the Serrania de Perija on the border with Venezuela. *Calamagrostiscrispifolius* differs from all other species of *Calamagrostis* s.l. by the presence of strongly curled, readily deciduous leaf blades, amongst numerous other characteristics including open inflorescences with generally patent branches, small spikelets, (3.5–)4–5.5 mm long, with sessile florets and a rachilla prolongation reaching from 2/3 to almost the apex of the lemma, with short hairs (< 1 mm long). *Deschampsiasantamartensis* is similar to *Deschampsiahackelii* (=*Calamagrostishackelii*) from austral South America but differs by its broad, rigid and erect, strongly conduplicate blades, 1.5–2.5 mm wide when folded, ligules of innovations 0.5–1 mm long, truncate or obtuse, ligules of upper flowering culms 3–4 mm long, broadly shouldered with an attenuate central point, ellipsoid spike-like panicle, 3–5.5 long × 1.5–2.5 cm wide, lemma surfaces moderately to lightly scabrous between the veins, lemma apex acute to muticous, entire, rachilla extension often absent and inside of the floret often with hyaline shiny sinuous trichomes to 1 mm long, emerging from the base of the ovary. We also present a broader circumscription of the common species *Deschampsiapodophora* (=*Calamagrostispodophora*), with the new variety D.podophoravar.mutica described and illustrated. Deschampsiapodophoravar.mutica principally differs from var. podophora by florets lacking awns and larger habit i.e. multiple taller culms with longer and wider leaf blades forming tussocks, with inflorescences often held within sheaths. Nomenclatural changes are presented, with *Deyeuxiamacrostachya* newly synonymised under *C.macrophylla* and *C.pittieri*, *C.pubescens* and *Deyeuxiapubescens* newly synonimised under *C.planifolia*. Lectotypes are designated for *Agrostisantoniana*, *Calamagrostispisinna*, *Deyeuxiamacrostachya* and *Deyeuxiasodiroana*. We also document and give notes on five new records of *Calamagrostis* for Colombia: *C.carchiensis*, *C.guamanensis*, *C.heterophylla*, *C.pisinna* and *C.rigida*.

## Abstract (es)

*Calamagrostis* (syn. *Deyeuxia*), como traditionalmente está delimitado, es uno de los géneros con mayor número de especies registradas para las zonas paramunas de Sudamérica y el sur de Centroamérica, en donde a menudo es un elemento dominante en hábitats de alta montaña tropical. Sin embargo, es aun difícil para investigadores identificar con precisión las especies de este género, principalmente por la falta de tratamientos florísticos para la mayoría de países que contienen páramo, con la distribución de muchas especies todavía poca conocida. Con el fin de realizar un aporte al conocimiento de las gramíneas tropicales, se presenta una lista actualizada de nombres y claves de identificación taxonómica en inglés y español (como appendice electrónico) de las especies de *Calamagrostis* s.l. conocidas o que probablemente se encuentran en los páramos de Perú, Ecuador, Colombia, Venezuela, Costa Rica y Panamá. Como resultado, 54 especies son aceptadas, de las cuales 47 especies están circunscritas en *Calamagrostis* y siete especies están recientemente transferidas a *Deschampsia*. Como novedades taxonómicas, se presentan dos nuevas especies, *Calamagrostiscrispifolius* y *Deschampsiasantamartensis*, las cuales están descritas e ilustradas. Estas nuevas especies crecen en páramos de la Sierra Nevada de Santa Marta (departamento de Magdalena), en la punta más al norte de Colombia, con *C.crispifolius* también presente en la Serranía de Perijá en la frontera con Venezuela. *Calamagrostiscrispifolius* difiere de las otras especies de *Calamagrostis* s.l. por la presencia de láminas foliares fuertemente crispadas y fácilmente caducas, entre otras características incluyendo inflorescencias abiertas con ramas generalmente patentes, espiguillas pequeñas, (3.5–)4–5.5 mm long., con antecios sésiles y una prolongación de la raquilla llegando desde 2/3 hasta casi el ápice de la lema, con tricomas cortos (< 1 mm long.). *Deschampsiasantamartensis* es parecida a *Deschampsiahackelii* (=*Calamagrostishackelii*) de Sudamérica austral, pero difiere por sus láminas foliares siendo anchas, rígidas y rectas, y fuertemente conduplicadas, 1.5–2.5 mm de ancho cuando plegada, lígulas de las innovaciones 0.5–1 mm long., truncadas u obtusas, lígulas de la parte superior de las cañas floríferas 3–4 mm long., anchas con un punto central atenuado, panojas elipsoides y espiciforme, 3–5.5 long. × 1.5–2.5 cm de ancho, superficies de las lemas moderada a levemente escabroso entre las venas, ápices de las lemas agudas a múticas, enteras, prolongación de la raquilla a menudo ausente, y la presencia de tricomas hialinos, brillantes, y sinuosos de hasta 1 mm long., los cuales salen de la base del ovario. Por último, se presenta una circunscripción más amplia de la especie común *Deschampsiapodophora* (=*Calamagrostispodophora*), con la nueva variedad D.podophoravar.mutica, la cual es descrita e ilustrada. Deschampsiapodophoravar.mutica difiere principalmente de la var. podophora porque en sus antecios las aristas están ausentes, y su hábito es más grande i.e. con múltiples cañas largas con láminas foliares más largas y anchas, con inflorescencias a menudo escondidas dentro de las vainas. Los cambios nomenclaturales que se presentan son *Deyeuxiamacrostachya* sinonimizada bajo *C.macrophylla*, y *C.pittieri*, *C.pubescens* y *Deyeuxiapubescens* sinonimizada bajo *C.planifolia*. Designamos los lectotipos para *Agrostisantoniana*, *Calamagrostispisinna*, *Deyeuxiamacrostachya* y *Deyeuxiasodiroana*. También documentamos y damos notas de cinco nuevos registros de *Calamagrostis* para la flora de Colombia: *C.carchiensis*, *C.guamanensis*, *C.heterophylla*, *C.pisinna*, y *C.rigida*.

## Introduction

Páramos are vast, mesic, grass-dominated ecosystems found above the treeline in northwest South America and southern Central America, with their distribution stretching more-or-less continuously along the Andes from northern Peru to Venezuela, while also being found as more isolated ‘islands’ in Costa Rica and Panama (Luteyn 1999; Peyre et al. 2018). These grasslands are considered to be one of the world’s biodiversity hotspots hosting exceptional levels of diversity and endemism, but are facing unprecedented habitat degradation and loss (Myers et al. 2000; Pérez-Escobar et al. 2018). Despite grasses being the dominant ecosystem-modulating component of páramos (Rangel-Churio 2000, 2010), they remain poorly studied within these ecosystems, with Ecuador, Colombia and Venezuela still lacking floristic treatments to the cool-season grasses, Pooideae and there only being a few genera that have received attention (e.g. *Festuca* L.; Stančík and Peterson 2007).

*Calamagrostis* Adans. is considered one of the most speciose genera of páramo grasses, with 35 (Laegaard 2005) to 37 species reported for páramos (Luteyn 1999) and the only other genus with possibly larger number of species found in the páramo being *Festuca* with 56 species (Stančík and Peterson 2007). It is also considered to be one of the most dominant genera in páramo grasslands, with certain species such as *Calamagrostiseffusa* (Kunth) Steud. dominating biomass (Rangel-Churio 2000, 2010; Peyre et al. 2018). Taxonomic research focussed on *Calamagrostis* from páramos is limited to various country or regional checklists (Colombia: Giraldo-Cañas 2011, 2013; Giraldo- Cañas et al. 2016. Ecuador: Jørgensen and Ulloa Ulloa 1994; Jørgensen and León-Yánez 1999. Venezuela: Hokche et al. 2008; Bono 2010. Costa Rica: Morales Quirós 2003), with Luteyn (1999) providing a list of species for all páramos. Species descriptions and a means to differentiate species have been limited to original protologues and revisions from Costa Rica (Morales Quirós 2003), Peru (Tovar 1993), a synoptic treatment for Venezuela (Briceño 2010), as well as Hitchcock’s (1927) synoptic treatment of grasses from Peru, Ecuador and Bolivia. Unfortunately, countries with the highest proportion of páramo, Ecuador, Colombia and Venezuela, still lack revisions of *Calamagrostis*.

The taxonomy of *Calamagrostis* has been disputed over a long period, with many authors placing South American species in *Deyeuxia* Clarion ex P. Beauv. (e.g. Rúgolo de Agrasar 2012). However, recent phylogenetic research does not support the separation of *Deyeuxia* s.s. from *Calamagrostis* s.s. (Saarela et al. 2010, 2017; Kellogg 2015; Soreng et al. 2015, 2017). The most recent phylogenetic research by Saarela et al. (2017) found *Calamagrostis* s.l. to be polyphyletic and highlighted that its circumscription needs reassessment. *Ammophila* Host was found to be nested within a large *Calamagrostis* s.s. clade, while other taxa of *Calamagrostis* and *Deyeuxia* were nested within other lineages of the broader Agrostidinae + Brizinae + Calothecinae clade (part of Calothecinae of Soreng et al. 2015 reapportioned to subtribe Echinopogoniniae, these collectively united in supersubtribe Agrostidodinae of Soreng et al. 2017) or the *Deschampsia* P. Beauv. clade of the Holcinae p.p. (Aristaveninae of Soreng et al. 2017), bringing the monophyly of the whole Agrostidinae into question (Saarela et al. 2017). Another group of Latin American, mainly South American, taxa placed in *Calamagrostis* or *Deyeuxia* align in the Koeleriinae subtribe (Saarela et al. 2017); Koeleriinae was included in Aveniinae s.l. by Soreng et al. (2017). A reticulate relationship, involving species of this set of *Calamagrostis*, Koeleriinae and the newly described African-Asian polyploid genus *Trisetopsis*, was detected by Wölk and Röser (2014, 2017).

Examples of this taxonomic upheaval can be seen in *Calamagrostiseffusa*, a widespread species dominating páramos of Colombia, Venezuela and Ecuador, that was found to be strongly supported as sister to *Chascolytrum* Desv. of the Calothecinae p.p., a genus with inflated multiflowered spikelets that bears little resemblance to *Calamagrostis* (Saarela et al. 2017). Similarly, the North American species *Calamagrostiscoarctata* Eaton is placed in a new, albeit weakly supported, clade alongside *Dichelachne* Endl., *Echinopogon* P. Beauv. (Agrostidinae p.p.) and *Relchela* Steud. (Echinopogonineae of Soreng et al. 2017), all with largely varying morphologies (Saarela et al. 2017), but often with pubescent ovaries (Soreng et al. 2017). Saarela et al. (2017) also found most, but not all, species of Calamagrostissect.Deyeuxiasubsect.Stylagrostis (Mez) Escalona to be placed in a clade with species of *Deschampsia* (Holcinae p.p.; Aristaveninae of Soreng et al. 2017), a polyploid genus of 30–40 species distributed in temperate regions of the northern and southern hemispheres (Kellogg 2015). *Deschampsia* has been traditionally separated from *Calamagrostis* by the presence of 2–3 florets per spikelet, amongst other characters, while taxa of Calamagrostissubsect.Stylagrostis usually have only one floret with an additional floret rarely found. Calamagrostissubsect.Stylagrostis was traditionally delineated from other members of *Calamagrostis* by the presence of an extended rachilla internode between the glumes and the floret, i.e. floret stipitate. However, placement of *Calamagrostispisinna* Swallen in a separate clade to that of *Deschampsia* taxa in analyses by Saarela et al. (2017) points towards multiple origins of the stipitate floret used to define Calamagrostissubsect.Stylagrostis (Saarela et al. 2017). Taxa, previously circumscribed in Calamagrostissubsect.Stylagrostis, which have been transferred to *Deschampsia*, all exhibit a combination of stipitate florets and lustrous, smooth glumes or sparsely scabrous on the glume keels, although these characteristics are also shared by taxa placed in other clades e.g. *C.pisinna*.

Although recent phylogenetic research, most notably that of Saarela et al. (2017), has shown *Calamagrostis*, as traditionally circumscribed, to be polyphyletic and that eventually many taxa will need to be transferred to other genera, ecologists, botanists and others interested in the natural history of the páramo need to know what species are present in these ecosystems and have the means to identify them. We present a guide to the species of *Calamagrostis* s.l., as traditionally circumscribed, from the páramos of northwest South America and southern Central America. We include identification keys in English and Spanish (as electronic appendix), an updated species list, describe and illustrate two new species and one new variety and give notes on five new records of *Calamagrostis* s.l. for Colombia.

## Methods

The species list and dichotomous keys cover all species of *Calamagrostis* s.l. listed in the most recent checklists for Colombia (Giraldo-Cañas 2011, 2013; Giraldo- Cañas et al. 2016), Ecuador (Jørgensen and Ulloa Ulloa 1994; Jørgensen and León-Yánez 1999), Venezuela (Hokche et al. 2008; Bono 2010), Costa Rica (Morales Quirós 2003), páramos in general (Luteyn 1999), species mentioned for these countries in the Catalogue of New World Grasses (Soreng and Greene 2003; Soreng et al. 2003 and onwards) and species that potentially occur but are so far recorded only from drier high-elevation Puna vegetation further south (Zulma Rúgolo de Agrasar, pers. comm.). Accepted species follow Soreng et al. (2003 and onwards). Excluded or ambiguous species are listed after the species list.

Type specimens and protologues were seen, where possible, for most of the species, with herbarium revision being done at AAU, COL, FMB, HUA, JUMA, K, UPTC and US. Floristic treatments were consulted from Argentina (Rúgolo de Agrasar 1978, 2006, 2012), Chile (Rúgolo de Agrasar 1978), Bolivia (Hitchcock 1927; Villavicencio 1995, 1998), Peru (Hitchcock 1927; Tovar 1993), Ecuador (Hitchcock 1927; Laegaard 1998), Venezuela (Dorr et al. 2000; Briceño 2010; Dorr 2014), Costa Rica (Pohl 1980; Morales Quirós 2003) and Central America (Pohl and Davidse 1994). Taxonomic treatments of Calamagrostissubsect.Stylagrostis (Escalona 1988a, b, 1991) were also consulted. Specimen localities are cited by country (uppercase letters), political region (also historically called ‘departamento’ in Colombia, ‘provincia’ in Ecuador and ‘estado’ in Venezuela; in bold in the ‘Specimens examined’ sections) and then (where possible) municipality. Coordinates are given in Degrees and Decimal Minutes (DDM).

In this treatment, glabrous means without pubescence (‘pubescence’ in the sense of slender, relatively soft hairs). Smooth indicates no prickle-hairs with broad bases and/or hooked or pointed apices (i.e. pubescence can occur on a smooth surface and a rough or scabrous surface can be glabrous). The term “florets being stipitate” refers to the floret (first, proximal floret in rare cases where two florets are present in a spikelet) being inserted on a distinctly elongated lowermost rachilla internode that is present above the glumes, while the term “florets being sessile” refers to the absence of the aforementioned elongated lowermost rachilla internode.

## Results

### Key to species of *Calamagrostis* s.l. known or likely to occur in the páramos of north-west South America and southern Central America

This key covers the 54 páramo species currently accepted (see ‘species list’ below). Species not included in the key can be found in the section ‘excluded or ambiguous species’ below. A Spanish version of the key, “Supplementary Key (in Spanish): Clave a las especies de *Calamagrostis* s.l. conocidas o que probablemente ocurrirán en los páramos de noroeste Sudamerica y el sur de Centroamerica”, can be found as an electronic appendix.

**Table:** 

1	Florets always stipitate (lowermost rachilla internode distinctly elongated between the glumes and the floret) (the first floret stipitate), stipe 0.4–4 mm long (sometimes a little shorter, to 0.2 mm, in *Deschampsiaparodiana*, *D.santamartensis*, *Calamagrostisboyacensis* and *C.ramonae*; so various spikelets should be checked), cylindrical, dilated towards its apex (can be seen at the base of the glumes after the floret has fallen); leaves with or without a ligular stipule present at the junction between the sheath and blade	**2**
–	Florets usually sessile (lowermost rachilla internode not, or not noticeably, prolonged between the glumes and the floret) (the first floret sessile), stipe less than 0.3 mm long; leaves without a ligular stipule present at the junction between the sheath and blade	**21**
2(1)	Florets lacking awns	**3**
–	Florets awned, awn straight or twisted and bent (cf. *Deschampsiaaurea* sometimes mucronate)	**4**
3(2)	Plants 9–20 cm tall, leaves forming a basal mat to 12 cm tall that is much shorter than the exerted culms; leaf blades short and broad, 2.5–8 cm long, 1.5–2.5 mm wide as folded, strongly conduplicate; inflorescence spike-like, 3–5.5 cm long, 1.5–2.5 cm wide; rachilla prolongation absent or small and glabrescent	*** Deschampsia santamartensis ***
–	Plants 26–110 cm tall, leaf blades both basal and cauline, forming medium sized tussocks with some cauline blades often surpassing the inflorescence; leaf blades of innovations and lower flowering culm 11–22 cm long, 0.35–1.2 mm wide when rolled or folded, narrow and conduplicate or filiform involute or convolute, rarely completely flat; leaf blades of upper flowering culm 2.9–25 cm long, 2–7 mm wide when opened out, flat, conduplicate or convolute towards the apices; inflorescence open to semi-contracted, 10–25 cm long, (3–)5–8 cm wide; rachilla prolongation present and with hairs from ¾ to the apex of the lemma	** Deschampsia podophora var. mutica **
4(2)	Inflorescences dense and spike-like, usually < 3 cm wide	**5**
–	Inflorescences open to semi-contracted, usually > 3 cm wide (*C.cleefii*, *C.guamanensis* and *C.pisinna* sometimes to 1 cm wide, but generally lax and few-flowered)	**13**
5(4)	Callus hairs usually < 1 mm long or absent, not reaching the middle of the lemma; awn bent, twisted, > 5 mm long	**6**
–	Callus hairs reaching from ½ the length to surpassing the apex of the lemma (sometimes shorter in *C.teretifolia* and *Deschampsiapodophora*); awn straight, < 5 mm long (twisted and bent, 5.5–8 mm long in *C.boyacensis*)	**8**
6(5)	Calluses glabrous; awn ca. 5 mm long; leaf blades glabrous, scabrous; lemma 3.7–5 mm long, apex bifid	***C.chaseae*** (in part)
–	Calluses pilose, hairs usually < 1 mm long; awn (5–)6–7(–8.8) mm long; leaf blades ciliate (*C.violacea*) or densely pilose (*C.mollis*); lemma 3.8–6 mm long, apex 4-dentate (*C.violacea*) or terminating in 4 thin setae (*C.mollis*)	**7**
7(6)	Anthers 0.4–0.6 mm long; lemma apex terminating in 4 thin setae; leaf blades densely pilose abaxially and adaxially	*** C. mollis ***
–	Anthers 1.6–2.2(–2.4) mm long; lemma apex 4-dentate; leaf blades glabrous on their surfaces but margin usually ciliate	*** C. violacea ***
8(5)	Awns 5.5–8 mm long, twisted and bent, clearly passing the glumes; anthers 0.5–0.7 mm long; spikelets (5–)6–7.5 mm long; rachilla hairs reaching the lemma apex or briefly surpassing it	*** C. boyacensis ***
–	Awns < 5.5 mm long, straight, not reaching to slightly passing the apices of the glumes; anthers 0.5–2.6 mm long (only 0.5–0.6 mm long in *D.ovata*, rarely 0.7 mm long in *D.podophora*); spikelets 3.5–14 mm long; rachilla hairs reaching from 2/3 to surpassing the apex of the lemma	**9**
9(8)	Inflorescences greenish-purple, often interrupted, oval to narrowly elliptic; spikelets 3.5–6 mm long; rachilla hairs reaching from 2/3 to almost the apex of the lemma	**10**
–	Inflorescences golden and shining, continuous and without interruption, oblong, ovoid, ellipsoid, subspherical, cylindrical or capitate; spikelets (5–)7–14 mm long; rachilla hairs usually reaching or surpassing the lemma apex, rarely shorter	**11**
10(9)	Inflorescences usually interrupted and with a lobulate outline, oval to elliptic, 10–25 cm long × 3–8 cm wide; glumes 3.5–5.5 mm long; lemma 2.4–3.5 mm long; callus hairs reaching from 1/3 the length of the lemma to almost the lemma apex; rachilla hairs reaching from 4/5 to almost the apex of the lemma	***Deschampsiapodophora*** (in part)
–	Inflorescences usually only slightly interrupted, narrow-elliptic, 5–6 cm long × ca. 1 cm wide; glumes ca. 6 mm long; lemma ca. 3.5 mm long; callus hairs ca. ½ the length of the lemma; rachilla hairs reaching from 2/3 to 4/5 the length of the lemma	*** C. teretifolia ***
11(9)	Anthers 0.5–0.6 mm long; glumes (6.2–)7.8–14 mm long; ligules (ligular stipules) acuminate, 6–18 mm long	*** Deschampsia ovata ***
–	Anthers 1–2.6 mm long; glumes 5–8 mm long; ligular stipules acuminate (*D.chrysantha*) or ligules with a bifid apex (*D.aurea*), 0.7–20 mm long	**12**
12 (11)	Florets generally ½ or < ½ the length of the glumes; anthers 0.9–1.6 mm long; callus hairs reaching from ½ to ¾ the length of the lemma; awn inserted in the upper 1/3 of the lemma but not surpassing the lemma apex or inserted in the lower 1/3 and surpassing the apex of the lemma, sometimes absent; ligular stipule inconspicuous (rarely completely absent in some leaves); ligules with a bifid apex, 0.7–20 mm long	*** Deschampsia aurea ***
–	Florets usually longer than ½ the length of the glumes; anthers 1.6–2.6 mm long; callus hairs almost reaching or surpassing the apex of the lemma; awn inserted in the lower 1/3 of the lemma, not surpassing the apex of the lemma or briefly surpassing it; ligular stipule conspicuous, hyaline, acuminate, with 2 conspicuous keels that fade towards the apex, (0.7–)7–20 mm long	*** Deschampsia chrysantha ***
13(4)	Awns straight or slightly curved, 1.5–4 mm long, usually not passing the apices of the glumes or passing them only briefly; spikelets more or less glomerate in the distal part of the inflorescence branches	**14**
–	Awns twisted and bent, > 5 mm long, easily passing the apices of the glumes (sometimes straight in *C.boyacensis* and *C.cleefii* but then > 5 mm long); spikelets not glomerate	**16**
14(13)	Anthers 0.4–0.5 mm long; rachilla 1–1.2 mm long, sparsely pilose with hairs usually not reaching the apex of the palea; inflorescences golden and shining, lax, with pendant branches and spikelets glomerate in the distal part of the branches; plants usually 50–80 cm tall	*** Deschampsia parodiana ***
–	Anthers (0.7–)1.2–2.5 mm long; rachilla (0.8–)1–2.5 mm long, sparsely pilose with hairs that usually do not reach the apex of the palea (*D.eminens*) or pilose with long hairs that reach the apex of the palea or lemma or amply pass them (*D.podophora*); inflorescences golden and shining (*D.eminens*) or greenish with purple tints (*D.podophora*), lax with pendant branches and spikelets glomerate in the distal part of the branches or semi-spikelike with spikelets from the base; plants 20–130 cm tall	**15**
15(14)	Inflorescences golden and shining, lax with pendant branches and spikelets clustered in distal or proximal glomerules of 1–2 cm diameter; anthers 1.6–2.5 mm long; rachillas (0.8–)1–1.8 mm long., sparsely pilose, with hairs that usually do not reach the apex of the palea; plants (36–)50–130 cm tall	*** Deschampsia eminens ***
–	Inflorescences greenish with purple tints, lax with pendant branches and spikelets glomerate in the distal part of the branches or semi-spikelike with spikelets from the base; anthers (0.7–)1.2–1.5(–1.9) mm long; rachillas (1.2–)1.4–2.5 mm long, pilose with hairs that reach the apex of the palea or lemma or amply pass them; plants 20–75(–110) cm tall	***Deschampsiapodophora*** (in part)
16(13)	Lemmas bifurcated with a deep cleft that reaches almost the middle of the lemma, the awn arising from the base of the deep cleft between the two lobes	**17**
–	Lemmas dentate, not noticeably bifurcate (i.e. with a deep cleft); the awn arising from the dorsal surface of the lemma and not from the base of a deep cleft in the lemma	**18**
17(16)	Junction between the leaf blade and the sheath swollen; leaf blades folded, 2–3 mm wide when opened out; keels of the glumes smooth; callus hairs reach or surpass ½ the length of the lemma; rachilla usually capitate, reaching to ¾ the length of the lemma or more, with hairs greatly surpassing the apex of the lemma	***C.guamanensis*** (in part)
–	Junction between the leaf blade and the sheath not swollen; leaf blades flat or folded, 1–5.2 mm wide when opened out; keels of the glumes scabrous; callus hairs do not reach more than 1/3 the length of the lemma; rachilla not capitate, usually reaching ½ the length of the lemma, with hairs usually only reaching the apex of the palea (reaching or passing the apex of the lemma in specimens from the east of Colombia)	***C.pisinna*** (in part)
18(16)	Hairs of the callus and rachilla twisted; anthers ca. 0.5 mm long; junction between the leaf blade and the sheath swollen; awn twisted (usually more than 2 times) and bent in the direction of the base of the lemma, ca. 5 mm long; keels of the glumes scabrous; plants 30–50 cm tall; leaf blades long and flat, 8–19 cm long, 3–4 mm wide, scabrous adaxially and abaxially	*** C. ramonae ***
–	Hairs of the callus and rachilla straight; anthers 1–2.4 mm long; junction between the leaf blade and the sheath not swollen; awn twisted and bent, briefly twisted at the base and curved or straight, facing outwards and away from the base of the lemma, (5–)5.4–8(–8.8) mm long; keel of the glumes smooth or scabrous; plants 10–30(–37) cm tall; leaf blades shorter, 1.5–11(–14) cm long, narrow or broad, convolute, subinvolute, conduplicate or flat, at least the adaxial surface smooth	**19**
19(18)	Lemmas (3.8–)4.4–5(–5.8) mm long; anthers 1.6–2.2(–2.4) mm long; awn twisted (usually more than two times) and bent, inserted 1–1.6 mm from the base of the lemma; keel of the glumes scabrous or ciliate; ligules (1.2–)1.6–3.2(–3.8) mm long	*** C. violacea ***
–	Lemmas 2.2–2.8 mm long; anthers 1–1.5 mm long; awn briefly twisted at the base and curved or straight, inserted close to the base of the lemma (*C.cleefii*) or in the middle third of the lemma (*C.boyacensis*); keel of the glumes smooth; ligules ca. 0.5 mm long (*C.cleefii*) or 5–17 mm long (*C.boyacensis*)	**20**
20(19)	Awn inserted close to the base of the lemma, ca. 5 mm long; ligules ca. 0.5 mm long.; leaf blades 2–5 cm long	*** C. cleefii ***
–	Awn inserted in the middle third of the lemma, 6–7 mm long; ligules 5–17 mm long; leaf blades 5–11 cm long	*** C. boyacensis ***
21(1)	Rachilla prolongation absent; callus rounded, not recurved; callus hairs reaching from ½ to ¾ the length of the lemma; lemma apex obtuse and briefly cleft; glumes lanceolate; panicles open; leaf blades flat	*** C. llanganatensis ***
–	Rachilla prolongation present, well-developed and pilose to short and glabrous (sometimes very short in e.g. *C.bogotensis*); callus rounded, acute or recurved; combination of the other characters mentioned above not present	**22**
22(21)	Callus hairs reaching from ½ to more than the length of the lemma; panicle lax and open, usually pyramidal, sometimes with very few spikelets	**23**
–	Callus hairs reaching up to 1/3 the length of the lemma; panicle lax and open to dense and spike-like	**27**
23(22)	Calluses recurved; lemmas 3.5–4 mm long; anthers 0.4–0.8 mm long; leaf blades flat or rarely subconvolute; plants 60–130 cm tall	**24**
–	Calluses rounded or rarely acute, not recurved; lemmas 2–3 mm long; anthers 0.8–1.5 mm long; leaf blades flat, conduplicate, convolute or involute; plants ca. 60 cm (*C.steyermarkii*) or 6–25 cm tall	**25**
24(23)	Callus hairs slightly shorter than the lemma, exceptionally reaching the lemma apex; the glumes only surpass the floret briefly, by 0.5–0.6 mm; awn inserted in the middle third of the lemma; ligules 1–4 mm long	*** C. rupestris ***
–	Callus hairs surpassing the lemma apex; the glumes amply surpass the length of the floret by 1.5–6 mm; awn inserted in the upper third of the lemma; ligules generally 1–1.5 mm long	*** C. viridiflavescens ***
25(23)	Plants forming tussocks ca. 65 cm tall; leaf blades convolute, 15–25 cm long; inflorescence ca. 18 cm long; ligules obtuse, 2–2.5 mm long; calluses with dense hairs reaching from ½ to ¾ the length of the lemma; floret sessile, lowermost rachilla internode not prolonged between the glumes and the floret	*** C. steyermarkii ***
–	Plants forming a short basal lawn 6–25 cm tall; leaf blades convolute, conduplicate or flat, 2–8 cm long; ligules truncate to obtuse, < 1mm long; calluses with dense hairs reaching from ½ the length of the lemma to surpassing the lemma apex; floret short-stipitate, lowermost rachilla internode prolonged between the glumes and the floret by 0.1–0.3(–1) mm	**26**
26(25)	Anthers 1.1(–1.5) mm long; awn ca. 5 mm long; callus hairs reaching the lemma apex; rachilla ca. 2 mm long, with hairs reaching the lemma apex	*** C. cleefii ***
–	Anthers 0.8–1 mm long; awn 7–9 mm long; callus hairs reaching half the length of the lemma; rachilla ca. 1.5 mm long, with hairs surpassing the lemma apex	***C.guamanensis*** (in part)
27(22)	Panicles lax and open, usually pyramidal, generally larger than 8 cm long, with verticillate or semi-verticillate branching, branches divergent, straight or flexuous, sometimes the lower contracted and pendulous or with lower branches distanced between each other and much longer than the upper, sometimes pendulous (*C.mandoniana*)	**28**
–	Panicles spikelike or semi-spikelike, fusiform, elliptical or capitate, lateral branches contracted, few- to many-flowered, frequently interrupted towards the base	**37**
28(27)	Awns suprabasal, inserted 0.3–0.6 mm from the base of the lemma; lemma 3–4 mm long	**29**
–	Awns basal or medial, inserted 1–2.5 mm from the base of the lemma; lemma 3–7 mm long	**30**
29(28)	Rachillas ca. 0.9 mm long, glabrescent; lemma apex bifid; awn equalling or shorter than the lemma, not surpassing the glumes; ligule 1.5–2 mm long	*** C. divergens ***
–	Rachillas 1.5–2 mm long, sparsely pilose with long hairs; lemma apex dentate with the lemma veins briefly excurrent; awn briefly surpassing the glumes; ligule oblong, > 4 mm long	*** C. naiguatensis ***
30(28)	Rachillas glabrous except for a few short hairs at the base that usually do not reach ½ the length of the lemma; glumes 5–6 mm long; lemma ca. 5 mm long; lemma apex diminutely bifid; awn ca. 4.5 mm long	*** C. scaberula ***
–	Rachillas pilose with short or long hairs that, in both cases, reach from ¾ the length of the lemma to surpassing the lemma apex; combination of the other characters mentioned above not present	**31**
31(30)	Rachilla prolongation (not including the hairs) reaching from ¾ to almost the apex of the lemma, generally with hairs < 1 mm long, that usually do not reach the lemma apex; awns (2–)4.5–7 mm long; glumes 3–4(–5) mm long; lemmas 3–3.9(–4.8) mm long, lemma apex truncate, irregularly dentate or bidentate; inflorescence branches and pedicels usually smooth, rarely scaberulous; inflorescence branches patent; anthers 2–2.3 mm long	**32**
–	Rachilla prolongation (not including the hairs) up to ½ the length of the lemma (sometimes up to 2/3 in *C.macrophylla* and *C.planifolia*), with hairs > 1 mm long, reaching or briefly passing the lemma apex in *C.mandoniana* and *C.planifolia* or reaching the apex of the palea in *C.macrophylla*, *C.pisinna* and *C.secunda* (*C.planifolia* sometimes with hairs reaching only 2/3 but then has (1–)2 anthers); awns 5–9 mm long; glumes 3–8.2 mm long; lemmas 3.4–6.2 mm long; lemma apex cleft, 2-lobed, or denticulate; inflorescence branches and pedicels scaberulous or scabrous; inflorescence branches flexuous, slightly divergent (pendulous in *C.mandoniana*); anthers 0.8–2.8 mm long	**33**
32(31)	Leaf blades strongly curled, forming a basal mat to 20 cm tall in mature plants and much shorter than half the length of the flowering culms; leaf blades readily deciduous at maturity and covering the ground around the tufts	*** C. crispifolius ***
–	Leaf blades straight, forming tussocks 40–60(–107) cm tall with the leaves greater than half the length of the flowering culms; leaf blades not obviously deciduous	*** C. effusa ***
33(31)	Lemmas bifurcated with a deep cleft that reaches almost the middle of the lemma, the awn arising from the base of the deep cleft between the two lobes; anthers 2(–3), 0.9–1 mm long; spikelets 3.5–5(–6) mm long	***C.pisinna*** (in part)
–	Lemmas not strongly bifurcate (i.e. with a deep cleft); the awn arising from the dorsal surface of the lemma and not from the base of a deep cleft in the lemma; anthers 2 (*C.planifolia*) or 3; spikelets 3–8.2 mm long	**34**
34(33)	Anthers 2, rarely 1, 1–1.4(–2) mm long; glumes (3–)3.6–6 mm long; lemma 3.4–4.5(–5.5) mm long; lemma apex 2-lobed, lobes bidentate; basal leaf blades generally flat, 1.8–7 mm wide	***C.planifolia*** (in part)
–	Anthers 3, 2–2.8 mm long; glumes (5–)5.8–8.2 mm long; lemma (4.4–)5–7 mm long; lemma apex briefly cleft and 4-dentate (*C.mandoniana*), bifid with aristulate teeth (*C.macrophylla*) or bifid and 4-dentate (*C.secunda*); basal leaf blades usually cylindrical or subcylindical, involute or convolute, sometimes the upper blades opening to become flat (*C.macrophylla*, *C.mandoniana*)	**35**
35(34)	Rachilla hairs usually reaching the lemma apex or briefly passing it; inflorescence (24–)28–52 cm long, with pendulous branches; lower branches 10–16 cm long; lemma apex cleft and 4-dentate; ligules 2-12 mm long; leaf blades flat or cylindrical and involute, ca. 3.3–3.6 mm wide when opened out	*** C. mandoniana ***
–	Rachilla hairs reaching from ½ to 4/5 the length of the lemma, usually not passing the apex of the palea; inflorescence 15–30 cm long, with divergent to ascending branches; lower branches usually < 10 cm long; lemma apex bifid, often with aristulate teeth; ligules 3–6 (possibly longer?) mm long; leaf blades cylindrical or subcylindical, involute or convolute, sometimes the upper blades opening out to become flat	**36**
36(35)	Upper leaf blades often opening out to become flat, generally wide, 2–4 mm wide, involute in the lower portion of the plant; ligule bifid, with the segments lanceolate, smooth, 3–6 (possibly longer?) mm long; lemmas 5–7 mm long, apex bifid with aristulate teeth	*** C. macrophylla ***
–	Leaf blades generally cylindrical or subcylindical, involute or convolute, ca. 2 mm wide when opened out; ligule subtrigonous, slightly scabrous, ca. 4 mm long; lemmas ca. 4.7 mm long, apex bifid with 4 teeth but without aristulate teeth	*** C. secunda ***
37(27)	Anthers 0.2–0.6 mm long, generally adherent to the apex of the caryopsis, flowers generally cleistogamous (NB. *C.jamesonii* and *C.curta* usually chasmogamous but anthers measure 0.4–0.6 mm long); lemma apex terminating in 4 aristulas or 4 awned or erose deltoid teeth; stylopodium well-developed, 0.4–0.6 mm long (stylopodium absent or brief in *C.curta* and *C.jamesonii*)	**38**
–	Anthers > 1 mm long, free, flowers chasmogamous; lemma apex cleft or erose, 2–4-dentate, teeth regular or irregular, not awned; stylopodium not differentiated, brief or incipient if present	**48**
38(37)	Rachillas sparsely pilose or glabrous, if pilose the hairs short, indistinct, not reaching past the middle of the lemma	**39**
–	Rachillas with long well-developed hairs reaching from 2/3 to the apex of the lemma (*C.curta* and *C.sclerantha* have shorter hairs that reach from ½ to ¾ the length of the lemma)	**45**
39(38)	Awn straight or slightly curved, inserted in the middle third of the lemma, generally not reaching past the apices of the glumes; flowering culms rigid, thickened, slightly curved, with inflorescence generally subincluded in the flag leaf sheath; rachilla glabrous or glabrescent	*** C. rigescens ***
–	Awn twisted and bent, inserted in the lower third of the lemma, surpassing the apices of the glumes; flowering culms slender, erect and straight, with inflorescences generally exerted; rachilla sparsely pilose with short hairs, hairs sometimes found only at the apex	**40**
40(39)	Leaf blades, at least those of the flowering culms, flat, tender and glabrous (or rarely with ciliate margins)	**41**
–	Leaf blades all convolute or folded, junciform, rigid, curved or flexuose **43**
41(40)	Lemmas (2.2–)3–3.5(–4.2) mm long, usually scabrous throughout; leaf blades heteromorphic, those of the flowering culms flat, tender and glabrous (or rarely with ciliate margins), usually wider than the blades of the tillers, which are usually convolute, pilose on both sides or only with the margen pilose	*** C. heterophylla ***
–	Lemmas (4.5–)5–7 mm long, smooth at least at the base (*C.brevipaleata* scabrous towards the apex); leaf blades all flat, isomorphic (*C.hirta*) or heteromorphic but not differentiated in width and size but only in that the blades of the tillers are pilose while those of the flowering culm are glabrous (*C.brevipaleata*)	**42**
42(41)	Lemmas smooth throughout; leaf blades isomorphic, those of the flowering culm and those of the tillers similar, flat and pilose, 1–11 cm long	*** C. hirta ***
–	Lemmas smooth proximally and scabrous towards their apices; leaf blades heteromorphic, the blades of the flowering culm are glabrous while those of the tillers are villous, 10–25 cm long	*** C. brevipaleata ***
43(40)	Plants 1–5 cm tall, forming dense cushions; leaf blades obtuse, < 1 cm long, curved against the ground; inflorescences exerted, formed of 3–10 spikelets	*** C. minima ***
–	Plants (1.5–)4–50 cm tall, forming lax mats, not dense cushions; leaf blades conduplicate, acute or navicular, straight or curved but not prostrate against the ground; inflorescences exerted or subincluded in the sheaths, formed of few or many spikelets	**44**
44(43)	Leaf blades filiform, 0.2–0.4 mm wide when opened out, involute, curved or flexuose, exceptionally straight; rachilla 0.4–0.9(–1.2) mm long, sparsely pilose with hairs to 1.3 mm long that usually do not reach ½ the length of the lemma; lemma (2.6–)3.1–5.8 mm long	*** C. vicunarum ***
–	Leaf blades > 1 mm wide when opened out, folded, usually straight; rachilla (0.8–)1–2 mm long, with hairs usually 0.5–1 mm long that barely reach ½ the length of the lemma; lemma 4.5–5 mm long	*** C. fibrovaginata ***
45(38)	Glume keels ciliolate	*** C. jamesonii ***
–	Glume keels scaberulous to scabrous	**46**
46(45)	Lemma apex with 4 scabrous aristulas 1–2.1 mm long, that equal or pass the apices of the glumes or at least the upper glume	*** C. setiflora ***
–	Lemma apex with 4 membranaceous deltoid teeth 0.2–0.5(–0.7) mm long, not aristulate, shorter than the glumes	**47**
47(46)	Flowers cleistogamous; stylopodium present, 0.5–0.6 mm long; inflorescence 1.7–5 cm long, with many spikelets, sub-spikelike; glumes 4.5–5.2 mm long, 1-veined, exceptionally the upper glume 3-veined	*** C. sclerantha ***
–	Flowers chasmogamous; stylopodium absent; inflorescence to 2 cm long, with few spikelets, subglobose; glumes 4.8–6.5 mm long, the lower 1-veined, the upper 3-veined	*** C. curta ***
48(37)	Florets awnless	*** C. ecuadoriensis ***
–	Florets awned, awn dorsal or subapical	**49**
49(48)	Rachillas with long dense hairs that generally equal or surpass the lemma apex, exceptionally only reaching the apex of the palea in *C.planifolia* and *C.rigida* (*C.planifolia* sometimes with hairs reaching only 2/3 but can be distinguished by having only (1–)2 anthers)	**50**
–	Rachillas with short hairs or glabrous, if pilose then the hairs generally reaching to 4/5 the length of the lemma, but not reaching the apex of the palea (N.B. *C.macrophylla* sometimes has hairs reaching the apex of the palea)	**55**
50(49)	Leaf blades generally flattened, becoming convolute upon drying, 2–7 mm wide; androecium formed of (1–)2 stamens; anthers 0.8–1.4(–2) mm long	***C.planifolia*** (in part)
–	Leaf blades permanently involute or strongly conduplicate for their entire length; androecium formed of 3 stamens; anthers (1.8–)2–3 mm long	**51**
51(50)	Leaf blades recurved or slightly arching, 1–5(–9) cm long; leaves forming a basal mat shorter than half the length of the flowering culms; plants 5–35(–50) cm tall, forming small to medium sized mats, sometimes forming lax or dense cushions; inflorescences generally < 5 cm long; awn not surpassing the apex of the glumes	*** C. spicigera ***
–	Leaf blades rigid, erect, tough, sharply pointed, (6–)10–30 cm long; leaves not forming a notable basal mat, usually forming tussocks greater than half the length of the flowering culms (*C.killipii* can be variable); plants (0.15–)0.3–1.3 m tall, generally forming medium or large tussocks; inflorescences (5–)9–30 cm long (5–8 cm in *C.killipii*); awn not surpassing the apex of the glumes or greatly exerted from the glumes	**52**
52(51)	Inflorescences 5–8 cm long; plants small, < 30 cm tall; ligules ca. 0.8 mm long	*** C. killipii ***
–	Inflorescences (8–)9–20(–30) cm long; plants generally large, (15–)23–100 cm tall; ligules (1–)1.5–15 mm long	**53**
53(52)	Inflorescences sub-spikelike, erect, oval in outline, with tinges of gold and bronze, branches contracted, densely flowered from the base, sometimes lobulate and subnutant; leaf blades conduplicate, 1–1.4 mm wide as folded; ligule coriaceous to chartaceous, with an obtuse to acute apex, 1–10 mm long	*** C. glacialis ***
–	Inflorescences flexuose, greenish or violaceous, discontinuous, branches more or less contracted, generally lacking spikelets proximally; leaf blades convolute or conduplicate, ca. 1 mm wide as folded; ligules membranous, acuminate, generally 8–15 mm long	**54**
54(53)	Lemmas (4.2–)4.5–5.2(–5.4) mm long; awn 4–5.8 mm long, reaching the apex of the glumes or briefly passing it; rachilla with hairs that reach from ¾ the length of the lemma to the lemma apex	*** C. rigida ***
–	Lemmas (5–)5.4–6.2(–6.6) mm long; awn 5.4–7.4 mm long, amply surpassing the apex of the glumes; rachilla with hairs that amply surpass the lemma apex, exceptionally equalling it	*** C. intermedia ***
55(49)	Awns 0.7–4.5(–5) mm long, included within or scarsely passing the glumes, delicate and straight to slightly twisted (bent and emerging from between the glumes in *C.scabriflora* and *C.chaseae*); rachilla and callus glabrous or sparsely pilose, the hairs to 0.8 mm long and not reaching ½ the length of the lemma	**56**
–	Awns 5.5–9 mm long, exerted from the glumes, twisted and/or sometimes bent; rachilla usually with hairs that reach from ½ to ¾ the length of the lemma or the apex of the palea (hairs sometimes short in *C.involuta* and *C.fuscata* but awns measure > 5.5 mm long)	**60**
56(55)	Awns inserted in the lower third of the lemma; anthers 1	**57**
–	Awns inserted in the middle or above the middle of the lemma; anthers 1–2 (*C.carchiensis*, possibly *C.chaseae*) or 3	**58**
57(56)	Awns inserted 0.3–0.7 mm from the base of the lemma, (2.3–)3–4 mm long, straight or slightly bent	*** C. bogotensis ***
–	Awns inserted ca. 1 mm from the base of the lemma, ca. 1.7 mm long, bent and emerging from between the side-margins of the glumes	*** C. scabriflora ***
58(56)	Awns 0.7–1 mm long; anthers 1 (rarely 2 in material from eastern Colombia)	*** C. carchiensis ***
–	Awns ca. 3–5 mm long; anthers 3 (possibly 1 in *C.chaseae*, although further revision needed)	**59**
59(58)	Calluses scarcely pilose with short hairs ca. 0.1 mm long; floret sessile; awn ca. 3 mm long, inserted 3/5 from lemma base	*** C. fulgida ***
–	Calluses glabrous; floret stipitate, with the lowermost rachilla internode prolonged between the glumes and the floret by ca. 0.2 mm; awn ca. 5 mm long, inserted medially	***C.chaseae*** (in part)
60(55)	Leaf blades curved, recurved, flexuose or straight; plants with leaves forming a basal mat generally shorter than half the length of the flowering culms; mats small or medium sized with flowering culms 11–32 cm tall; inflorescences generally 5–12 cm long; rachilla sparsely pilose, the hairs usually reaching the middle of the lemma and not passing the apex of the palea	**61**
–	Leaf blades rigid, erect, tough, sharply pointed; plants forming dense tussocks with leaves greater than half the length of the flowering culms, 10–180 cm tall; inflorescences 9–31 cm long; rachilla with hairs reaching from ½ to ¾ the length of the lemma or apex of the palea	**62**
61(60)	Awns inserted in the lower third of the lemma; rachilla 1.3–1.4 mm long, sparsely pilose with short hairs or almost glabrous; anthers 0.7–1 mm long; lemma 3–4.1 mm long; old basal sheaths fibrous; ligules 0.5–1.5 mm long; glumes 5–5.7 mm long	*** C. involuta ***
–	Awns inserted in the middle third of the lemma; rachilla (1.4–)1.6–2.5 mm long, lightly pilose with hairs not reaching the apex of the palea; anthers ca. 1.5 mm long; lemma (3.8–)4–4.6 mm long; old basal sheaths not fibrous; ligules 1.2–2(–2.5) mm long; glumes 5.6–6.2 mm long	*** C. fuscata ***
62(60)	Upper leaf blades opening to become flat, generally wide, 2–4 mm wide, involute in the lower portion of the plant; panicle slightly open	*** C. macrophylla ***
–	Leaf blades convolute or conduplicate for their entire length, 0.3–2 mm wide as folded; panicle dense (*C.recta*) or slightly open (*C.tarmensis*)	**63**
63(62)	Upper glumes 3-veined, lateral veins brief, reaching to 1/3 the full length; inflorescence erect, sub-spikelike, lateral branches short and appressed; leaf blades scabrous, erect, sharply pointed, rigid; glumes 5.4–8(–8.5) mm long	*** C. recta ***
–	Upper glumes 3-veined, lateral veins longer, surpassing ½ the full length (shorter in C.tarmensisvar.tarijensis); inflorescence with lateral branches somewhat flexuose; leaf blades erect and rigid with the adaxial surface scabrous or scabrous-pubescent or blades somewhat flexuose with the adaxial surface scaberulous (C.tarmensisvar.tarijensis); glumes (4.4–)4.8–6.2(–7) mm long	*** C. tarmensis ***

### Species list of Calamagrostis s.l. known and likely to occur in the páramos of northwest South America and southern Central America

Fifty-four species of *Calamagrostis* s.l., constituting 47 species currently circumscribed in *Calamagrostis* and seven species currently circumscribed in *Deschampsia*, are here accepted as known or likely to occur in the páramos of northwest South America. Compared to the previous checklist of páramo taxa (Luteyn 1999) that documented 37 species, we accept 21 additional species (marked below with an asterisk (*), although two of these still need verification [*Calamagrostisglacialis* (Wedd.) Hitchc., *C.mandoniana* (Wedd.) Pilg.], while placing four species accepted by Luteyn (1999) as synonyms [*Calamagrostisnuda* (Pilg.) Pilg., *C.coarctata* (Kunth) Steud., *C.pittieri* (Kunth) Trin. ex Steud., *C.weberbaueri* Tovar].

Accepted species are in **bold**. Species which are not mentioned in the previous páramo checklist (Luteyn 1999) are marked with an asterick (*). Excluded or ambiguous species are listed and discussed below. Notable basionyms or synonyms are included. Countries where the species is recorded are shown with the following codes: AR=Argentina, BO=Bolivia, CHI=Chile, CO=Colombia, CR=Costa Rica, EC=Ecuador, MEX=Mexico, PAN=Panama, PE=Peru, VE=Venezuela. Countries where the species is likely to occur, but so far has not been recorded, are noted with a question mark (?). Information on the elevational range is taken from relevant literature, herbarium specimen revision and field observations.

***Calamagrostisbogotensis*** (Pilg.) Pilg.; basionym: *Deyeuxiabogotensis* Pilg.; syn.: *Calamagrostisnuda* (Pilg.) Pilg., *Deyeuxianuda* Pilg.; distr.: CO, CR, EC, PAN?, PE, VE; alt.: 2300–4400 m.

***Calamagrostisboyacensis*** Swallen & García-Barr.; syn.: *Calamagrostisweberbaueri* Tovar, *Deyeuxiaweberbaueri* (Tovar) Rúgolo ex Luteyn; distr.: CO, EC, PE; alt.: 3000–4800 m.

*Calamagrostisbrevipaleata Swallen; distr.: EC; alt.: 2800–5000 m.

***Calamagrostiscarchiensis*** Lægaard; distr.: COL, EC; alt.: 3200–3900 m.

***Calamagrostischaseae*** Luces; distr.: VE; alt.: 3000–4000 m.

***Calamagrostiscleefii*** Escalona; distr.: CO, EC; alt.: 3300–4500 m.

*Calamagrostiscrispifolius Sylvester; distr.: CO, VE; alt.: 2700–3570 m.

*Calamagrostiscurta (Wedd.) Pilg.; basionym: *Deyeuxiacurta* Wedd.; distr.: AR, BO, EC; alt.: 4200–5000 m.

***Calamagrostisdivergens*** Swallen; distr.: CO; alt.: 3100–3200 m.

***Calamagrostisecuadoriensis*** Lægaard; distr.: EC; alt.: 3450–4500 m.

***Calamagrostiseffusa*** (Kunth) Steud.; basionym: *Deyeuxiaeffusa* Kunth; syn.: *Calamagrostisareantha* (Pilg.) Pilg., *Calamagrostisfunckii* Steud., *Deyeuxiaaraeantha* Pilg., *Deyeuxiaareantha* Pilg., *Deyeuxiafunckii* Steud. ex Wedd.; distr.: CO, EC, VE; alt.: 3000–4700 m.

***Calamagrostisfibrovaginata*** Lægaard; basionym: *Deyeuxiacoarctata* Kunth; syn.: *Calamagrostiscoarctata* (Kunth) Steud. [nom. illeg., hom., blocked by *Calamagrostiscoarctata* Eaton]; distr.: CO, EC, PE, VE; alt.: 2500–4700 m.

*Calamagrostisfulgida Lægaard; distr.: EC; alt.: 2500 m.

*Calamagrostisfuscata (J. Presl) Steud.; basionym: *Deyeuxiafuscata* J. Presl.; distr.: BO, EC, PE; alt.: 3900–4500 m.

*Calamagrostisglacialis (Wedd.) Hitchc.; basionym: *Deyeuxiaglacialis* Wedd.; distr.: BO, EC?, PE; alt.: 3900–5000 m.

***Calamagrostisguamanensis*** Escalona; distr.: COL, EC; alt.: 3300–4400 m.

***Calamagrostisheterophylla*** (Wedd.) Pilg.; basionym: *Deyeuxiaheterophylla* Wedd.; syn.: *Calamagrostiscalvescens* Pilg., *Calamagrostismacbridei* Tovar; *Calamagrostismulleri* Luces; *Calamagrostisswallenii* Tovar, *Deyeuxiaswallenii* (Tovar) Rúgolo; distr.: AR, BO, CHI, COL, EC, PE, VE; alt.: 3100–4650 m.

***Calamagrostishirta*** (Sodiro) Lægaard; basionym: *Deyeuxiahirta* Sodiro; distr.: EC; alt.: 2900–4600 m.

***Calamagrostisintermedia*** (J. Presl) Steud.; basionym: *Deyeuxiaintermedia* J. Presl; syn.: *Calamagrostisagapatea* Steud. ex Lechl.; distr.: BO, CO, CR, EC, MEX, PAN, PE; alt.: 2400–5000 m.

***Calamagrostisinvoluta*** Swallen; distr.: CO; alt.: 3700–3800 m.

***Calamagrostisjamesonii*** Steud.; syn: *Calamagrostisstuebelii* (Pilg.) Pilg., *Deyeuxiajamesonii* (Steud.) Munro ex Wedd., *Deyeuxiastuebelii* Pilg.; distr.: BO, CO, EC, PE, VE; alt.: 3600–4900 m.

***Calamagrostiskillipii*** Swallen; distr.: CO, VE; alt.: 3500–4200 m.

***Calamagrostisllanganatensis*** Lægaard; distr.: EC; alt.: 3500–4000 m.

***Calamagrostismacrophylla*** (Pilg.) Pilg.; basionym: *Deyeuxiamacrophylla* Pilg.; syn.: *Deyeuxiamacrostachya* Sodiro; distr.: CO, EC, PE; alt.: 3000–4000 m.

*Calamagrostismandoniana (Wedd.) Pilg.; basionym: *Deyeuxiamandoniana* Wedd.; distr.: BO, PE; alt.: 3200–4100 m.

*Calamagrostisminima (Pilg.) Tovar; basionym: Calamagrostisvicunarumvar.minima Pilg.; syn.: *Deyeuxiaminima* (Pilg.) Rúgolo; distr.: AR, BO, EC, PE; alt.: 4300–4600 m.

***Calamagrostismollis*** Pilg.; syn: *Deyeuxiamollis* (Pilg.) Sodiro; distr.: EC, PE?; alt.: 3200–5100 m.

*Calamagrostisnaiguatensis Swallen; distr.: VE; alt.: 2300–2800 m.

***Calamagrostispisinna*** Swallen; distr.: CO, VE; alt.: 3200–4400 m.

***Calamagrostisplanifolia*** (Kunth) Trin. ex Steud.; basionym: *Deyeuxiaplanifolia* Kunth; syn.: *Calamagrostispittieri* Hack.; *Calamagrostispubescens* (Pilg.) Pilg.; distr.: BO, CO, CR, EC, PE, VE; alt.: 2600–4400 m.

***Calamagrostisramonae*** Escalona; distr.: EC, VE; alt.: ca. 3950 m.

***Calamagrostisrecta*** (Kunth) Trin. ex Steud.; basionym: *Deyeuxiarecta* Kunth; syn.: *Calamagrostishumboldtiana* Steud., *Calamagrostispallens* (J. Presl) Steud., *Deyeuxiapallens* J. Presl, *Deyeuxiastricta* Kunth, *Deyeuxiasulcata* Wedd.; distr.: BO, CO, EC, MEX, PE, VE; alt.: 2700–4900 m.

***Calamagrostisrigescens*** (J. Presl) Scribn.; basionym: *Agrostisrigescens* J. Presl; syn.: *Calamagrostisbromidioides* (Griseb.) Pilg., *Calamagrostiscajatambensis* Pilg., *Calamagrostisimberbis* (Wedd.) Pilg., *Deyeuxiacajatambensis* Pilg. ex Zuloaga, Nicora, Rúgolo, Morrone, Pensiero & Ciald., *Deyeuxiaimberbis* Wedd., *Deyeuxiarigescens* (J. Presl) Türpe; distr.: AR, BO, CHI, EC, MEX, PE; alt.: 3300–4600 m.

***Calamagrostisrigida*** (Kunth) Trin. ex Steud.; basionym: *Deyeuxiarigida* Kunth; syn.: *Calamagrostisantoniana* (Griseb.) D.M. Moore, *Calamagrostisantoniana* Steud., *Calamagrostiscrassifolia* Hack. ex Sodiro, *Calamagrostisgracilis* (Wedd.) Henrard, *Calamagrostisgracilis* (Wedd.) Pilg., *Calamagrostisgusindei* Pilg. ex Skottsb., *Calamagrostissandiensis* Pilg., *Deyeuxiaantoniana* (Griseb.) Parodi, *Deyeuxiacrassifolia* Sodiro, *Deyeuxiagracilis* Wedd., *Deyeuxiagusindei* (Pilg.) Parodi; distr.: AR, BO, CHI, COL, CR, EC, PAN?, PE, VE?; alt.: 2900–4900 m.

*Calamagrostisrupestris Trin.; syn.: *Calamagrostisbeyrichiana* Nees ex Döll, *Calamagrostislongearistata* (Wedd.) Hack. ex Sodiro, Calamagrostismontevidensisvar.linearis Hack., *Deyeuxiabeyrichiana* (Nees ex Döll) Sodiro, Deyeuxiaheterophyllavar.elatior Wedd., *Deyeuxialongearistata* Wedd., *Deyeuxiarupestris* (Trin.) Rúgolo; distr.: AR, BO, CO, EC, PE, VE, widespread in eastern South America; alt.: 700–3400 m.

*Calamagrostisscaberula Swallen; distr.: EC; alt.: ca. 2900 m.

*Calamagrostisscabriflora Swallen; distr.: VE; alt.: ca. 2500 m.

*Calamagrostissclerantha Hack.; syn.: *Calamagrostisspiciformis* Hack., *Deyeuxiasclerantha* (Hack.) Rúgolo, *Deyeuxiaspiciformis* (Hack.) Türpe; distr.: AR, BO, EC, PE; alt.: 2500–4600 m.

*Calamagrostissecunda (Pilg.) Pilg.; basionym: *Deyeuxiasecunda* Pilg. ; distr.: EC; alt.: ca. 4000 m.

*Calamagrostissetiflora (Wedd.) Pilg.; basionym: *Deyeuxiasetiflora* Wedd.; syn.: *Calamagrostiscoronalis* Tovar; distr.: AR, BO, EC, PE; alt.: 3600–5000 m.

***Calamagrostisspicigera*** (J. Presl) Steud.; basionym: *Deyeuxiaspicigera* J. Presl; syn.: *Deyeuxiaobtusata* Wedd., *Deyeuxiasubsimilis* Wedd.; distr.: AR, BO, CHI, CO, EC, PE; alt.: 3600–5000 m.

***Calamagrostissteyermarkii*** Swallen; distr.: EC; alt.: 3400–4200 m.

***Calamagrostistarmensis*** Pilg.; syn: *Calamagrostistarijensis* Pilg., *Deyeuxiatarmensis* (Pilg.) Sodiro; distr.: AR, BO, EC, PE; alt.: 2400–4700 m.

***Calamagrostisteretifolia*** Lægaard; distr.: EC; alt.: 4300–4900 m.

***Calamagrostisvicunarum*** (Wedd.) Pilg.; basionym: *Deyeuxiavicunarum* Wedd.; syn.: *Calamagrostispentapogonodes* Kuntze, *Calamagrostispulvinata* Hack., Calamagrostisspiciformisvar.acutifolia Hack. ex Buchtien, *Deyeuxiapulvinata* (Hack.) Türpe; distr.: AR, BO, CHI, CO?, EC, PE, VE; alt.: 3200–4900 m.

*Calamagrostisviolacea (Wedd.) Hack. ex Buchtien; basionym: *Deyeuxiaviolacea* Wedd.; syn.: *Calamagrostisviolacea* (Wedd.) Hitchc.; distr.: AR, BO, EC, PE; alt.: 4000–4900 m.

*Calamagrostisviridiflavescens (Poir.) Steud.; basionym: *Arundoviridiflavescens* Poir.; syn.: *Calamagrostissplendens* (Brongn.) Steud., *Calamagrostisviridescens* (Poir.) Steud., *Deyeuxiasplendens* Brongn., *Deyeuxiaviridiflavescens* (Poir.) Kunth; distr.: AR, BO, CHI, CO, EC, MEX, PE, widespread in eastern South America; alt.: 800–2500 m.

***Deschampsiaaurea*** (Munro ex Wedd.) Saarela; basionym: *Deyeuxiaaurea* Munro ex Wedd.; syn.: *Calamagrostisaurea* (Munro ex Wedd.) Hack. ex Sodiro, *Stylagrostislongigluma* (Pilg.) Mez; distr.: EC, PE?; alt.: 2900–4900 m.

*Deschampsiachrysantha (J. Presl) Saarela; basionym: *Deyeuxiachrysantha* J. Presl; syn.: *Calamagrostischrysantha* (J. Presl) Steud., *Stylagrostischrysantha* (J. Presl) Mez, *Stylagrostisleiopoda* (Wedd.) Mez; distr.: AR, BO, CHI, CO?, EC?, PE, VE; alt.: 3500–5000 m.

***Deschampsiaeminens*** (J. Presl) Saarela; basionym: *Deyeuxiaeminens* J. Presl; syn.: *Calamagrostiseminens* (J. Presl) Steud., *Stylagrostiselegans* (Wedd.) Mez, *Stylagrostiseminens* (J. Presl) Mez; distr.: AR, BO, CO, CHI, PE; alt.: 3600–4500 m.

*Deschampsiaovata (J. Presl) Saarela; basionym: *Deyeuxiaovata* J. Presl; syn.: *Calamagrostisovata* (J. Presl) Steud., *Calamagrostispflanzii* Pilg., *Deyeuxiaanthoxanthum* Wedd., *Deyeuxiacapitata* Wedd., *Deyeuxianivalis* Wedd., *Stylagrostisnivalis* (Wedd.) Mez, *Stylagrostisovata* (J. Presl) Mez; distr.: BO, EC, PE; alt.: 4000–5200 m.

***Deschampsiaparodiana*** Saarela; basionym: *Deyeuxialigulata* Kunth [name blocked by *Deschampsialigulata* (Stapf) Henrard]; syn.: *Calamagrostisligulata* (Kunth) Hitchc.; distr.: CO, EC, PE, VE; alt.: 3700–4850 m.

*Deschampsiapodophora (Pilg.) Saarela; basionym: *Calamagrostispodophora* Pilg.; syn.: *Deyeuxiapodophora* (Pilg.) Sodiro; distr.: CO, EC, PE, VE; alt.: 3500–4100 m.

*Deschampsiasantamartensis Sylvester & Soreng; distr.: CO; alt.: 4300–4500 m.

### Excluded or ambiguous species

#### Calamagrostis meridensis

Taxon classificationPlantaePoalesPoaceae

(Luces) B. Briceño, Bot. Ecol. Monocot. Páramos Venezuela. 2: 590. 2010. Agrostis meridensis Luces, Bol. Soc. Venez. Ci. Nat. 15(80): 11. 1953.

##### Type.

VENEZUELA. Mérida: coleccionado en el bosque de la Laguna Negra, Páramo de Muchuchies, alt.: 3500 m, 25 Nov. 1943, Zoraida Luces de Febres 267 (holotype: VEN; isotype: MO (MO1086043! [image!]) fragm. ex VEN)

##### Comments.

Briceño (2010) proposed the new combination of *Calamagrostismeridensis* for a taxon endemic to páramos of Venezuela. Briceño (2010) transferred the species from *Agrostis* to *Calamagrostis* based on the upper glume having 2–3 veins and anatomical characters such as all vascular bundles having a double sheath and without a notably angular shape and short cells over the veins can be solitary, in pairs or in short series. However, the species habit is noted as long rhizomatous to stoloniferous with geniculate culms, a habit unknown in *Calamagrostis* or *Deschampsia*, which are tufted or very-short rhizomatous and tussock-forming. This taxon is most likely a species of *Podagrostis* (Griseb.) Scribn. & Merr. based on the small spikelets (2.6–3.8 mm long), palea subequal to the lemma, awn often lacking, a rachilla extension that is usually absent or, when present, very short and glabrescent, a callus glabrous or rarely with scarce short hairs and short anthers 0.7–1 mm long. The number of veins of the upper glume has been considered as a distinguishing character to differentiate *Podagrostis* from *Calamagrostis* (e.g. Rúgolo de Agrasar 2012) but the recent discovery of *Podagrostiscolombiana* Sylvester & Soreng (Sylvester et al. in press) from the Colombian Andes with well-developed lateral veins of the upper glume, large anthers and tussock-forming habit has shown these characters to be labile in *Podagrostis*.

#### Deyeuxia sodiroana

Taxon classificationPlantaePoalesPoaceae

Hack. ex Sodiro, Revista Col. Nac. Vicente Rocafuerte 12: 64. 1930. Calamagrostis sodiroana Hack., Anales Univ. Centr. Ecuador 3(25): 481. 1889, nom. nud.

##### Type.

ECUADOR. Pichincha: Crece en los pajonales de los montes Pichincha, Chimborazo y El Altar [occid. M. Pichincha, Tablahuasi], Aug. 1888, L. Sodiro 25/10 (**lectotype, designated here**: W (W1916-0037841 [image!]); isolectotype: W (W1916-0037840 [image!])).

##### Comments.

Known only from the type collected in Ecuador, *Calamagrostissodiroana* likely belongs to *Deschampsia* sensu Saarela et al. (2017), earlier to *Calamagrostis sect. Deyeuxia* subsect. Stylagrostis due to the rachilla internode being extended between the glumes and floret. This species bears affinities to *Deschampsiaparodiana* and *Deschampsiapodophora* due to its open panicle and straight awn that usually does not or only slightly surpasses the glumes and blades usually much shorter than the flowering culms (Sodiro 1930: 70; Zulma Rúgolo de Agrasar, pers. comm.).

#### “ Calamagrostis

Taxon classificationPlantaePoalesPoaceae

sp. A” (Dorr 2014: 221).

##### Comments.

This taxon was first noted as “*Calamagrostischaseae* auct., non Luces” by Dorr et al. (2000 [2001]: 56) and subsequently called “*Calamagrostis* sp. A” in the Flora of Guaramacal (Venezuela): Monocotyledons (Dorr 2014) but its identity remains ambiguous and needs further study. The species habit is noted as stoloniferous, a habit not known from *Calamagrostis* or *Deschampsia*, which are tufted or very-short rhizomatous and tussock-forming. The mention of short spikelets (3–4.4 mm long), florets (appearing to) almost reach or equal the length of the glumes and 1-veined upper glumes in the brief description by Dorr (2014: 221) makes it possible that this taxon belongs to *Podagrostis* or *Agrostis*, although mention of a puberulent callus and presence of a fairly long awn (3.5–6 mm long) makes it less likely that this species is a *Podagrostis*.

#### Calamagrostis spruceana

Taxon classificationPlantaePoalesPoaceae

(Wedd.) Hack. ex Sodiro, Gram. Ecuator. (Anal. Univ. Quito) 3(25): 481. 1889. Deyeuxia spruceana Wedd., Bull. Soc. Bot. France 22: 178, 180. 1875. Deyeuxia toluccensis Munro ex Wedd., Bull. Soc. Bot. France 22: 180. 1875, nom. inval.

##### Type.

ECUADOR. [without precise locality], 1859, Jameson s.n. [#59] (holotype: P (P00740364 [image!])).

##### Comments.

Known only from the type collected in Ecuador, the identity of this taxon remains ambiguous. *Calamagrostisspruceana* bears similarities to *C.macrophylla, C.secunda* and *C.macrostachya* due to its open inflorescence, short hairs of the callus, rachilla hairs not reaching the apex of the palea and awn inserted slightly above the middle of the lemma (Sodiro 1930: 70; Zulma Rúgolo de Agrasar, pers. comm.).

#### Deyeuxia pendula

Taxon classificationPlantaePoalesPoaceae

Sodiro, Revista Col. Nac. Vicente Rocafuerte 12: 65. 1930.

##### Type.

ECUADOR. Crece en las pajonales del Pichincha entre 3650 y 4200 m, Sodiro s.n. (not located).

##### Comments.

Known only from the type and the identity of this taxon remains ambiguous.

### Nomenclatural changes (new synonyms)

#### Calamagrostis planifolia

Taxon classificationPlantaePoalesPoaceae

(Kunth) Trin. ex Steud., Nomencl. Bot. (ed. 2) 1: 251. 1840. Deyeuxia planifolia Kunth, Nov. Gen. Sp. (quarto ed.) 1: 145. 1815[1816]. Arundo planifolia (Kunth) Poir., Encycl. 4: 707. 1816.

**Type.** PERU. In montanis Andinum Peruvianorum prope Guangamarca, 1250 hexap. [2286 m], 1833, Bonpland s.n. (holotype: P (P00729787 [image!]); isotype: BAA (BAA00001855 [image!]) fragm. ex P, P (P030106 [image!]) fragm., LE (LE-TRIN-1801.01!) fragm. ex Herb. Willd. 176).
= Deyeuxiapubescens Pilg., Bot. Jahrb. Syst. 25(5): 712. 1898, syn. nov. Calamagrostispubescens (Pilg.) Pilg., Bot. Jahrb. Syst. 42: 60. 1908, syn. nov. Type: COLOMBIA. [Crescit in monte ignivomo Pasto] Volcán de Pasto, 3400 m alt., 9 Dec. 1869, Stübel 389b (lectotype, designated by Vega and Rúgolo de Agrasar (2013: 28): BAA (BAA00000811 [image!]) fragm. ex B; isolectotype: US (US00406355!) fragm. ex B).
= Calamagrostispittieri Hack., Oesterr. Bot. Z. 52(3): 108. 1902, syn. nov. Type: COSTA RICA. Cerro de Buena Vista, pres du sommit, [prope cacumen, Valle du General], 3100 m alt., 19 Jan. 1891, Pittier s.n. Pl. Costaric. Exs. 3359 (holotype: W (W19160029198 [image!]); isotypes: B, BR (BR0000006865702 [image!]), BAA (BAA00000766 [image!]) fragm. ex B).

##### Comments.

*Calamagrostispittieri*, recorded for Costa Rica, Colombia and Venezuela (Morales Quirós 2003; Hokche et al. 2008; Giraldo-Cañas 2011, 2013; Giraldo-Cañas et al. 2016) is synonymised under *Calamagrostisplanifolia* as no satisfactory characteristics were found to separate the two. Both have spikelets with florets bearing a pilose rachilla extension with hairs reaching from ¾ to passing the apex of the lemma, an awn inserted in the upper half of the lemma, 2 anthers that usually are short, ca. 1–1.4 mm long and leaf blades that are usually flat (sometimes drying convolute) with variable indumentum.

*Calamagrostispubescens* was considered an endemic species to Colombia (Giraldo- Cañas 2013) and only known from the type specimen that was collected in southern Colombia from hills surrounding Pasto of departamento Narino. The B holotype was destroyed during World War II, with fragments only remaining at BAA and US. The BAA fragment consists of spikelets whose size and characteristics match *C.planifolia* (Zulma Rúgolo de Agrasar, pers. comm.), and the description of vegetative characteristics in the protologue also matches *C.planifolia* and so we consider *C.pubescens* a synonym of *C.planifolia*. Nevertheless, further study is needed for the *C.planifolia* complex in Colombia and there are certain crucial characters, such as number of stamens that need to be verified in these taxa. The *C.pubescens* BAA fragment lacked anthers (Zulma Rúgolo de Agrasar, pers. comm.) and Pilger (1898: 712) did not describe them.

#### Calamagrostis macrophylla

Taxon classificationPlantaePoalesPoaceae

(Pilg.) Pilg., Bot. Jahrb. Syst. 42: 60. 1908. Deyeuxia macrophylla Pilg., Bot. Jahrb. Syst. 25(5): 711–712. 1898.

**Type.** ECUADOR. Pinchincha: Verdecuchu, Aug. 1879 [7 Aug. 1870], A. Stübel 34 (lectotype, designated by Vega and Rúgolo de Agrasar (2013: 27): BAA (BAA00000810 [image!] fragm. ex B; isolectotypes: S (S-R-823 [image!]) fragm., US (US00153721!, US00133532!)).
= Deyeuxiamacrostachya Sodiro, Revista Col. Nac. Vicente Rocafuerte 12: 64. 1930, syn. nov. Type: ECUADOR. In pasq. M. Chimbarazo [En los pajonales de los montes Pichincha y Chimborazo], Nov. [Dec.] 1890, L. Sodiro 265 [s.n.] (lectotype, designated here: US (US00406351!); isolectotype: S (S-R-1460 [image!])).

##### Comments.

*Deyeuxiamacrostachya* is known only from the type specimen collected by Sodiro in páramo grasslands close to mount Pichincha and Chimborazo, Ecuador. We found no noticeable differences between this and Pilgers specimens. The large, lax panicle with semiverticillate branches, similar spikelet morphology including the lemma apex bifid with aristulate teeth and rachilla hairs only reaching the apex of the palea, support *D.macrostachya* being synonymised under *C.macrophylla*.

### Two new species and a revised description and new variety of *Deschampsiapodophora*

Both of the new species are found in the páramos of Sierra Nevada de Santa Marta, at the northernmost tip of Colombia, with *Calamagrostiscrispifolius* also being found in the Sierra de Perija, on the border between Venezuela and Colombia, suggesting that these two high elevation environments, although fairly isolated from each other, share certain floristic affinities. The discovery of *Deschampsiasantamartensis* adds a further endemic grass species to the Sierra Nevada de Santa Marta alongside *Podagrostiscolombiana* Sylvester & Soreng (Sylvester et al. in press), and *Agrostopoawallisii* (Mez) P. M. Peterson Soreng & Davidse and *A.barclayae* Davidse, Soreng & P. M. Peterson, of the only endemic genus of Colombia, *Agrostopoa* Davidse, Soreng & P.M. Peterson (Davidse et al. 2009), and highlights the necessity for further collecting expeditions to be made to this isolated massif on the northernmost tip of Colombia.

A number of Colombian specimens of *Deschampsispodophora* (= *Calamagrostispodophora*) were also discovered that lacked certain diagnostic characters of the species and prompted the more comprehensive circumscription of the species presented here that includes description of the new variety Deschampsiapodophoravar.mutica.

#### Calamagrostis crispifolius

Taxon classificationPlantaePoalesPoaceae

Sylvester sp. nov.

urn:lsid:ipni.org:names:60478840-2

Fig. 1

##### Type.

COLOMBIA. Magdalena: flanco occidental de la Sierra Nevada de Santa Marta, páramo, abundantisima, cubre gran parte del páramo, 3140 m alt., 29 Jan. 1959, R. Romero Castañeda 7141 (holotype: COL (COL000172001!); isotype: US (US01246667!)).

**Figure 1.**
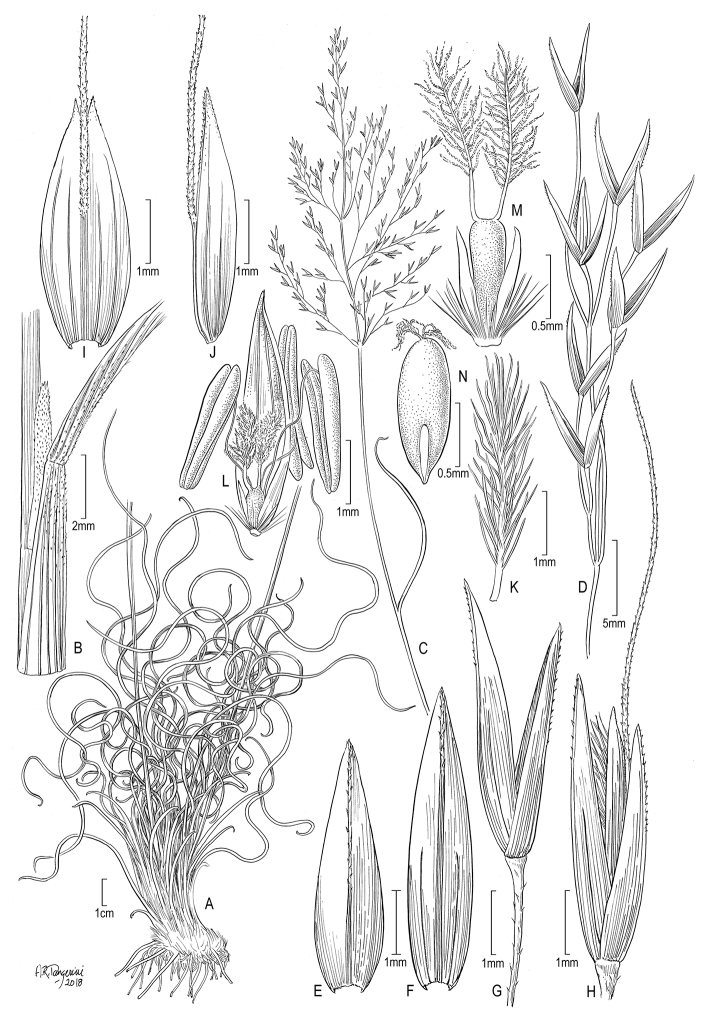
*Calamagrostiscrispifolius*; **A** lower portion of plant, showing basal tuft of curled leaves **B** lígular area **C** upper portion of plant showing inflorescence and flag leaf blade **D** primary branch of the inflorescence **E** lower glume, dorsal view **F** upper glume, dorsal view **G** spikelet, with floret already fallen, lateral view **H** spikelet, with floret, lateral view **I** lemma, dorsal view **J** lemma, lateral view **K** stylar branch and stigma **L** palea ventral view, showing the ovary, stamens and lodicules **M** ovary and lodicules, ventral view **N** immature caryopsis, dorsal view showing embryo; drawn by Alice R. Tangerini from the isotype, R. Romero Castañeda 7141 (US).

##### Diagnosis.

Differs from all other species of *Calamagrostis* s.l. by a combination of strongly curled, readily deciduous leaf blades in mature plants that form a basal mat to 20 cm tall, open inflorescences with generally patent branches, spikelets (3.5–)4–5.5 mm long, with sessile florets and a rachilla prolongation (not including hairs) reaching from 2/3 to almost the apex of the lemma, with short hairs < 1 mm long and an awn inserted just above the middle of the lemma, 5–7.2 mm long, anthers 1.5–2.7 mm long.

##### Description.

**Plants** perennial, tufted, forming short dense tufts, mats to 40 cm wide, with short vertical or oblique rhizomes. Bases covered with fibrous old basal sheaths, with fallen curled leaf blades often forming large masses on the ground between tufts. **Tillers** intravaginal. **Culms** 48–64 cm tall, 1.5–2.2 mm wide, striate, erect, greatly exerted from the basal foliage, nodes and internodes terete, smooth but becoming scabrous below the nodes and towards the inflorescence, with dense scabrocities just below the inflorescence; (0–)1 node exposed at flowering; uppermost internodes 38–42 cm long. **Sheaths** striate, moderately keeled; **flag leaf sheaths** 20–25 cm long; **upper culm sheaths** glabrous and smooth with minute papillae present on the adaxial surface; **basal leaf sheaths** 4–12 cm long, shorter than the internodes, glabrous and lightly scabrous. **Ligules** not stipulate; **upper culm ligules** 2.5–10 mm long, acute with a rounded or bidentate apex, scarious to coriaceous, 2-veined but without notable lateral keels, apices denticulate, fimbriate or short ciliate, ligule abaxial surface lightly to densely scabrous with short scabers; **ligules of innovations** 2.2–10 mm long, strongly decurrent with the sheaths, truncate to acute, scarious to coriaceous when shorter, lightly to densely scabrous on the abaxial surface. **Leaf blades** 5–15(–30) cm long, 0.5–1.5 mm wide, cylindrical in outline, when rolled the blades form a basal mat to 20 cm tall and much shorter than the exerted culms, strongly curled in mature plants [or when dry?], appearing readily deciduous and snapping off when the plant reaches maturity leaving an abscission zone and the ligule exposed, sometimes recurved to straight in immature plants [or when moist?], conduplicate to convolute, rigid, glabrous, abaxially finely scabrous, adaxially densely scabrous, edges smooth or slightly scaberulous, apex pungent; **flag leaf blade** ca. 2.9 cm long, recurved, slightly narrower than the basal blades. **Panicles** (5.5–)9–20 cm long, 3–10 cm wide, open, rarely contracted in young specimens, exerted or rarely subincluded in the flag leaf, open and diffuse, oval, greenish-purple or golden-purple; **main panicle axis** terete, glabrous, lightly to moderately scabrous, spikelets found diffusely on the proximal half of the primary branches, lower internodes 2–4 cm long; **panicle branches** spreading to slightly ascending, rarely contracted; **primary panicle branches** 2–6 cm long, bearing 1–10 spikelets per branch, verticillate in clusters of 2–5, terete, glabrous, almost smooth to scabrous; **pedicels** (3–)6–22 mm long, usually much longer than the spikelets, glabrous, lightly to moderately scabrous. **Spikelets** 1-flowered, not strongly laterally compressed, disarticulating above the glumes; glumes, lemma and palea not noticeably asymmetrical. **Glumes** (3.5–)4–5.5 mm long, subequal, the lower glume ca. 0.3 mm shorter than the upper glume, narrowly lanceolate, membranous, purplish, lustrous, dorsal surface smooth or scaberulous distally, keels lightly scabrous distally or scabrous throughout, apices acute, finely denticulate, erose; **lower glume** 1-veined; **upper glume** 3-veined, lateral veins reaching from ¼ to past half the length of the glume, 1 or 2 cross veins between the keel and lateral vein infrequently present in ca. 10% of spikelets seen (requires 50× magnification). **Floret** sessile, almost reaching the apex of the glumes, sometimes passing the apex of the lower glume. **Lowermost rachilla internode** not prolonged between the glumes and the floret. **Lemmas** 3.7–4.6 mm long, 5-veined, veins not evident; the same consistency as the glumes, golden, glabrous, scaberulous throughout or lustrous and faintly to densely muriculate with the apex sometimes becoming scaberulous, apex shortly emarginate with lobes finely denticulate, awns 5–7.2 mm long, amply passing the glumes, twisted at the base, slightly curved, densely scabrous throughout, inserted just above the middle of the lemma, at 2.2–2.5 mm from the lemma base. **Paleas** 0.3–0.8 mm shorter than the lemma, of the same consistency and colour, keels smooth and notable, apex bidentate. **Callus** rounded, short, articulation oblique, with a basal tuft of short hairs 0.2–0.7(–0.8) mm long. **Rachilla** (2–)3–3.5 mm long, reaching from 2/3 to almost the apex of the lemma, with copious short hairs 0.5–1 mm long, the hairs reaching from 4/5 to almost the apex of the lemma and usually surpassing the palea. **Lodicules** 2, 0.4–0.6 mm long, membranous, 2-lobed, acute to slightly acuminate. **Stamens** 3, anthers 1.5–2.7 mm long. **Ovary** ca. 0.5 mm long, small, styles 2, stigmas plumose with secondary branching, short. **Caryopsis** ca. 1.8 mm long, ca. 0.7 mm wide, elliptic, rounded triangular in transection, hilum narrowly elliptic, ventral groove shallow and not conspicuous, pale brown, embryo ca. 0.3 mm long, apex with remains of styles and stigmas; **endosperm** firm.

##### Distribution and ecology.

Colombia, Venezuela. Known from páramos of the Sierra Nevada de Santa Marta, northern Colombia and the Sierra de Perija, Venezuela. For the Sierra Nevada de Santa Marta, specimens are known from páramo vegetation on both the western and eastern flanks of the massif. For the Sierra de Perija, specimens are known from both the northern and southern extents of the mountain range. It is found growing between 2700–3570 m in páramos with dry soils that are often subject to fires. The plant forms dense cushion-like mats or clumps and it is a dominant component of certain páramos. The type specimen label includes “Abundantisima, cubre gran parte del páramo”, i.e. highly abundant and covers a large part of the páramo. The specimen label of Barclay and Juajibuoy 6545 (US) also states “the dominant grass on these slopes, extending up to the top of ridge”, thus implying that it is a dominant component of the páramos of the Sierra Nevada de Santa Marta.

The degree of curling of leaf blades in the different specimens studied may relate to the level of maturity of the plant or also the local microclimate, with the specimen label of Barclay and Juajibuoy 6545 (US) stating “leaves very fine and rolled, when dry they curl”. The characteristic of readily deciduous leaf blades is also interesting, with the specimen label of Barclay and Juajibuoy 6545 (US) also mentioning that “large masses of these [the fallen curled blades] on the ground between clumps [of the plant]”.

##### Other specimens examined.

COLOMBIA. **Magdalena**: Sierra Nevada de Santa Marta, Laguna Chubdula, 3480 m alt., 10°55'N; 73°53'W, 29 Dec. 1972, Kirkbridge & Forero 1775 (MO); Sierra Nevada de Santa Marta, alrededores de cabeceras del Río Sevilla, 3050–3300 m alt. [US specimen label states ‘The dominant grass on these slopes, extending up to the top of ridge. West and north facing slopes, on south side of river above campsite, sta.1,6., alt. 3320–3570 m’], 20 Jan. 1959, H.G. Barclay & P. Juajibioy 6545 (MO, US-3652630); Sierra de Perija, east of Manuare, Sabana Rubia, páramo, 3000–3100 m alt., 6 Nov. 1959, J. Cuatrecasas & R. Romero Castañeda 25021 (US [3 sheets]).

VENEZUELA. **Zulia**: Maracaibo Distr., Sierra de Perija, Serranía de Valledupar, Campamento Monte Viruela, on tepuí-like limestone massif 5×2.5 km in size, on the international boundary, 10°25.2167'N; 72°52.7'W, 3100 m alt., 25–28 Dec. 1974, S.S. Tillett 747-882 (MO); Perijá Distr., Sierra de Perija, Serranía de los Motilones, mesa below international boundary on main ridge, headwaters of Río Negro, Campamento Frontera II, 10°0.2167'N; 72°58.4167'W, 3000 m alt., 27 Nov.–5 Dec. 1974, S.S. Tillett & K.W. Hönig 746-618 (MO); Serranía de Valledupar, international boundary, headwaters of Río Guasare, 10°23.13'N; 72°52.0833'W, 2700–3300 m alt., 10–19 Dec. 1974, S.S. Tillett 747-1072 (MO).

##### Preliminary conservation status.

Vulnerable (VU). Despite the species being known from seven collections spanning two Cordilleras, the páramos of Colombia are currently facing threats principally from mining (Pérez-Escobar et al. 2018) and an uncertain future. Our preliminary conservation status of VU is deemed adequate until further research is done.

##### Etymology.

The species epithet refers to the strongly curled leaf blades which make it distinct from all other páramo taxa of *Calamagrostis* s.l. with open panicles.

##### Notes.

To our knowledge, there are no species of *Calamagrostis* s.l. with readily deciduous leaf blades that snap off when the plant reaches maturity leaving an abscission zone and the ligule exposed and covering the ground surrounding the plant tufts. This, coupled with the strongly curled nature of the leaf blades, makes this species unique. The character of curled leaf blades is very uncommon in the genus *Calamagrostis*, with the closest resembling species with this character being *Calamagrostiscrispa* (Rúgolo & Villav.) Soreng, a species found in dry Andean grassland of Bolivia, Chile, Peru and Northeast Argentina (Rúgolo de Agrasar 2012: 193). The blades of mature plants of *C.crispa* are generally curved rather than strongly curled, as in *C.crispifolius*. *Calamagrostiscrispa* also differs from *C.crispifolius* by the short, few-flowered, inflorescences that are included within the basal foliage, large spikelets with glumes 5–8 mm long and lemmas (4.4–)5–5.5 mm long, amongst other characters.

*Calamagrostiscrispifolius* also shares certain similarities with *C.effusa* in terms of characters of the inflorescence i.e. the open panicles with verticillate panicle branches, the short glumes to 5.5 mm long, the awn being inserted in the upper half of the lemma and, most importantly, the long rachilla usually extending past the apex of the palea and bearing short hairs < 1 mm long. *Calamagrostiscrispifolius* and *C.effusa* also share a peculiar character of cross veins between the lateral veins and keel of the upper glume, but these are only noticeable at 50× magnification in about 1 in 10 spikelets. A more exhaustive search for this character in other taxa within *Calamagrostis* s.l. should be done to check its exclusiveness to *C.crispifolius* and *C.effusa*. *Calamagrostiscrispifolius* can be easily distinguished from *C.effusa* by the strongly curled leaf blades with pungent apices which form a basal mat to 20 cm high that is usually much shorter than the flowering culms, while *C.effusa* has stiffly erect blades with obtuse apices forming tussocks 40–60(–107) cm tall. Ligule characteristics also differ, with *C.crispifolius* having ligules 2.2–10 mm long with acute apices, while *C.effusa* has ligules 1–2 mm long with truncate apices. In a recent molecular analysis, *Calamagrostiseffusa* was found to be sister to *Chascolytrum* Desv. (Saarela et al. 2017), possibly warranting its own generic placement. Further collecting of this new species, with a focus on molecular sampling, should be done to clarify its phylogenetic affinities.

Specimens of *C.crispifolius* from the Sierra Nevada de Santa Marta, Colombia, differ from Venezuelan specimens in a number of attributes and may represent a subspecies, although further collections and studies need to be made to confirm this. Colombian specimens have narrower leaf blades (0.5–0.75 mm wide), shorter ligules (2.2–4 mm long), usually longer anthers (to 2.7 mm long) and rachillas with short hairs that often reach the apex of the lemma. Venezuelan specimens have broader leaf blades (to 1.5 mm wide), longer ligules (4–10 mm long), shorter anthers (1.5–2 mm long) and rachillas with short hairs that usually do not reach the apex of the lemma.

#### Deschampsia santamartensis

Taxon classificationPlantaePoalesPoaceae

Sylvester & Soreng sp. nov.

urn:lsid:ipni.org:names:60478841-2

Fig. 2

##### Type.

COLOMBIA. Magdalena: Sierra Nevada de Santa Marta, valley descending south-western from Picos Reina and Ojeda, rocky and sandy páramos above Laguna Naboba and Laguna Reina, superpáramo, 4300–4500 m alt., 5 Oct. 1959, J. Cuatrecasas & R. Romero Castañeda 24607 (holotype: COL (COL000184738!); isotype: US (US01240776!)).

**Figure 2.**
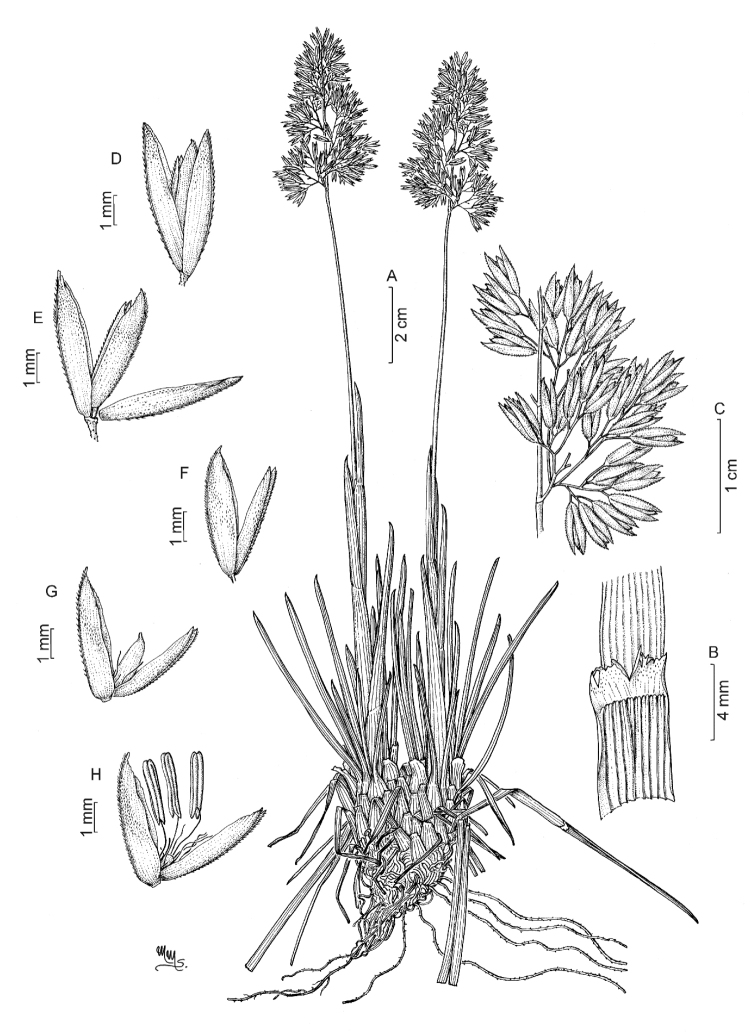
*Deschampsiasantamartensis*. **A** Habit **B** Ligule **C** Panicle branch **D** Spikelet **E** spikelet, with lower glume pulled away to reveal the stipitate floret (extension of the lowermost rachilla internode) **F** floret **G** floret, with palea and lemma slightly separated to reveal the ovary in a late stage of development and surrounded by long filamentous trichomes **H** floret, with palea and lemma slightly separated to reveal the stamens and ovary at an earlier stage of development and surrounded by long filamentous trichomes; drawn by Marcela Morales from the holotype, J. Cuatrecasas & R. Romero Castañeda 24607 (COL).

##### Diagnosis.

*Deschampsiasantamartensis* is similar to *Deschampsiahackelii* (Lillo) Saarela, but differs in having broad, rigid and erect, strongly conduplicate blades, 1.5–2.5 mm when folded (vs. filiform and curved leaf blades 0.5–1 mm wide when folded), ligules short and often truncate or obtuse with ligules of innovations 0.5–1 mm long and ligules of upper flowering culms 3–4 mm long (vs. ligules long and acuminate 3.5–7 mm long), ellipsoid spike-like panicles 3–5.5 long × 1.5–2.5 cm wide (vs. capituliform spherical panicles 1–3 cm long × 1–2 cm wide), lemma surfaces moderately to lightly scabrous between the veins (vs. smooth), lemma apices acute to muticous and entire (vs. truncate and irregularly dentate), rachilla extension often absent (vs. always present, 0.5–1 mm long) and inside of the floret often with hyaline shiny sinuous trichomes to 1 mm long emerging from the base of the ovary (vs. lacking trichomes).

##### Description.

**Tufted perennial** forming short dense tufts with vertical rhizomes and papyraceous, fibrous basal sheaths, leaves form a basal mat to 12 cm tall and much shorter than the exerted culms. **Tillers** intravaginal. **Culms** 9–20 cm tall, 1–1.5 mm wide, erect, exerted from the basal foliage, not obviously striate, densely papilliate; nodes and internodes terete, densely papilliate, not lustrous, nodes hidden in the sheaths with no nodes exposed at flowering; uppermost internodes 10–18 cm long, longer than the sheaths. **Sheaths** weakly striate, slightly keeled; **flag leaf sheaths** 7–10 cm long; **upper culm sheaths** lax, glabrous, smooth, densely papilliate; **basal leaf sheaths** 2–3 cm long, longer than the internodes, glabrous, smooth or slightly scabrous, densely papilliate, older basal sheaths papyraceous and fibrous with the fibres becoming slightly curly as the sheaths decay and leave just the fibres. **Ligules** not stipulate; **upper culm ligules** 3–4 mm long, regularly decurrent with the sheaths, broadly shouldered with an attenuate central point, hyaline, without notable lateral keels, apices entire or irregularly dentate, abaxial surface smooth and sparsely papilliate; **ligules of innovations** 0.5–1 mm long, strongly decurrent with the sheaths, broadly shouldered and sometimes with a lateral extension or lobe that extends ca. 1 mm long, the centre of the ligule truncate to obtuse and irregularly dentate, hyaline, without notable lateral keels, abaxial surface scabrous with distinct, mostly retrorse, spinules, with spinules tending to run down the throat and occur on the collar margin. **Leaf blades** 2.5–8 cm long, 1.5–2.5 mm wide when folded, gradually reduced up the culm, strongly conduplicate with margins frequently inrolled, weakly keeled, erect to slightly divergent, glabrous, abaxial blade surface smooth, strongly papilliate, adaxial blade surface scabrous mainly in the centre in lines along the veins, veins pronounced, margins moderately scaberulous, apex naviculate with a slightly pungent mucronate point; **flag leaf blade** 1.7–3 cm long, slightly narrower than lower culm blades. **Panicles** 3–5.5 cm long, 1.5–2.5 cm wide, dense, ellipsoid, golden-purple, densely spiculate, sometimes interrupted towards the base, spikelets present from near the base; **main panicle axis** terete, glabrous, smooth, lower internode ca. 1 cm long; **panicle branches** short, ascending; **primary panicle branches** 1–2 cm long, bearing 15–50 spikelets per branch, verticillate in clusters of 1–3, terete, glabrous, almost smooth to lightly scabrous, papilliate; **pedicels** 0.5–1.5 mm long, much shorter than the spikelets, glabrous, lightly scabrous, papilliate. **Spikelets** 1-flowered, strongly laterally compressed, disarticulating above the glumes (not seen); glumes, lemma and palea slightly asymmetrical. **Glumes** 4.6–5.5 mm long, subequal or rarely unequal, lower glumes 0.5–1.5 mm shorter than the upper glumes, membranous, purplish grading to a scarious golden-bronze margin, sub-lustrous, papilliate, very sparsely scabrous on the keels, edges smooth, apices acute to acuminate, entire to infrequently irregularly denticulate; **lower glume** 1-veined; **upper glume** 3 or sometimes faintly 5-veined, lateral veins reaching half the length of the glume. **Florets** stipitate, single, included in the glumes, subequalling the apex of the lower glume. **Lowermost rachilla internode** (0.25–)0.4–0.5 mm long, prolonged between the glumes and the floret, terete, slightly dilated at its apex, glabrous, smooth. **Lemmas** 4–4.5 mm long, of the same consistency as the glumes, golden-purple with a broad scarious margin and apex, glabrous, long scabrous to pectinate scabrous along the keel for most its length, lemma surface moderately to sparsely scabrous between the veins distally for up to ¾ the length of the lemma, apex slightly falcate, acute to muticous, entire, apex margins broadly scarious and somewhat incurved, 5-veined, veins generally not evident, sometimes apparent towards the base; awn absent. **Paleas** slightly shorter than the lemma, of the same consistency, golden, scarious, keels closely spaced with the keel flanges twice as broad as the gap between the keels, one keel more pronounced than the other, regularly scabrous and notable in the upper 2/3 of the length, scabrous between the keels, apex sometimes inconspicuously bidentate. **Callus** base rounded and slightly dorsally compressed above, glabrous. **Rachilla** absent or to 0.6(–1) mm long, glabrescent with a few short hairs at the apex. **Lodicules** 2, ca. 0.4 mm long, as broad as long, membranous, flabellate, with an acute lobe. **Stamens** 3, anthers 2–2.2 mm long. **Ovary** ca. 0.6 mm long, small, styles 2, stigmas plumose with secondary branching, short, often with scarce hyaline, shiny and sinuous trichomes to 1 mm long emerging from the base of the ovary. **Caryopsis** ca. 2 mm long, ca. 0.6 mm wide, obovate, laterally compressed in cross section, hilum 0.2 mm long, circular to obovate, ventral groove shallow and narrow and not conspicuous, pale honey brown, embryo ca. 0.4 mm long, apex with remains of style bases < 0.1 mm long, with remains of short plumose stigmas attached; **endosperm** liquid.

##### Distribution and ecology.

Known only from the type specimen that was collected from a southwest facing valley descending from Picos Reina and Ojeda in the centre of the Sierra Nevada de Santa Marta. The specimen was collected from high elevation rocky and sandy superpáramo vegetation above 4300 m.

##### Preliminary conservation status.

Data Deficient (DD). Currently known only from a single specimen. Further expeditions are needed to the Sierra Nevada de Santa Marta to document its distribution.

##### Etymology.

The species epithet refers to the type locality of the Sierra Nevada de Santa Marta.

##### Notes.

*Deschampsiasantamartensis* clearly belongs to Calamagrostissubsect.Stylagrostis (=*Deschampsia* sensu Saarela et al. 2017) due to the presence of an extended rachilla internode between the glumes and the floret. *Deschampsiasantamartensis* can be easily distinguished from all other members of Calamagrostissubsect.Stylagrostis with dense, spike-like panicles by its florets lacking awns, a glabrous callus and the absence of a rachilla extension (or rarely with a diminutive glabrescent rachilla extension). The only other member of Calamagrostissubsect.Stylagrostis with dense spike-like panicles, glabrescent callus, glabrescent short rachilla extensions and which occasionally lack awns is *Deschampsiahackelii* (Lillo) Saarela (= *Calamagrostishackelii* Lillo), a species known from high-Andean regions of northwest Argentina and Chile (Rúgolo de Agrasar 2012: 201). *Deschampsiahackelii* differs by its capituliform panicles, 1–3 cm long × 1–2 cm wide (vs. ellipsoid spike-like panicles 3–5.5 long × 1.5–2.5 cm wide), filiform and curved leaf blades 0.5–1 mm wide when folded (vs. broad, rigid and erect, strongly conduplicate blades 1.5–2.5 mm when folded), long acuminate ligules 3.5–7 mm long (vs. ligules short and often truncate or obtuse with ligules of innovations 0.5–1 mm long and ligules of upper flowering culms 3–4 mm long), lemma surfaces smooth (vs. moderately to lightly scabrous between the veins), lemma apex truncate and irregularly dentate (vs. acute to muticous and entire), rachilla extension always present 0.5–1 mm long (vs. often absent) and inside of the floret lacking trichomes (vs. often with hyaline shiny sinuous trichomes to 1 mm long emerging from the base of the ovary).

*Deschampsiaaurea*, a species originally described from Ecuadorian páramos but which also occurs in Peruvian Jalca vegetation, is another member of Calamagrostissubsect.Stylagrostis that has dense, spike-like panicles and spikelet lemmas that occasionally lose the thin weak dorsally-inserted awn (Zulma Rúgolo de Agrasar, pers. comm.). *Deschampsiaaurea* has well-developed callus and rachilla hairs, with callus hairs surpassing more than half the length of the lemma and rachilla hairs reaching or surpassing the lemma apex, as well as other characters to help differentiate it from *D.santamartensis* such as the floret being noticeably shorter (2.9–3 mm long) than the large glumes (5.5–7.5 mm long), amongst other characters. *Calamagrostischrysostachya* (E. Desv.) Kuntze, a species known from high-Andean regions of northwest Argentina and Chile, also shares these attributes and has also been included as a member of Calamagrostissubsect.Stylagrostis (Rúgolo de Agrasar 2012, pers. comm.). Both these species, however, have a pilose callus and rachilla, with *C.chrysostachya* having poorly developed callus and rachilla hairs to 1 mm long, but thin conduplicate or convolute leaf blades 0.8–1.2 mm wide when unfolded and a ligular stipule 0.5–1.5 mm long. The short, strongly conduplicate leaf blades with navicular apices are also reminiscent of *Poatrachyphylla* Hack. which also inhabits high-elevation superpáramo of Colombia (Sylvester et al. in press).

#### Deschampsia podophora

Taxon classificationPlantaePoalesPoaceae

(Pilg.) Saarela, PhytoKeys 87: 90. 2017. Deyeuxia podophora (Pilg.) Sodiro, Rev. Col. Nac. Vicente Rocafuerte 11: 79. 1930. Calamagrostis podophora Pilg., Bot. Jahrb. Syst. 42 (1): 66. 1908.

##### Type.

PERU. Junín: Berge Westlich von Huacapistana [Prov. Tarma, in montibus prope Huacapistana ad occid. in stepposis], 3500 m alt., 18 Jan. 1903, A. Weberbauer 2231 (lectotype, designated by Vega and Rúgolo de Agrasar (2013: 28): BAA (BAA00000767 [image!]) fragm. ex B; isolectotype: US (US00149282!)).

##### Description.

**Tufted perennial** with vertical rhizomes forming short solitary tufts to medium-sized tussocks, with leaf blades mostly basal with inflorescences usually greatly exerted from basal foliage or both basal and cauline with some cauline blades often surpassing the inflorescence. **Tillers** intravaginal. **Culms** 20–75(–110) cm tall, to 3 mm wide, erect, striate, nodes and internodes terete, smooth and lustrous; nodes hidden in the sheaths with no nodes exposed at flowering; uppermost internodes 20–32.5 cm long, as long or longer than the sheath. **Sheaths** striate; **flag leaf sheaths** 22–38 cm long; **upper culm sheaths** lax, glabrous and smooth; **basal leaf sheaths** 4.5–20 cm long, longer than the internodes, glabrous and smooth. **Ligules** not stipulate; **upper culm ligules** 7.5–22 mm long, strongly decurrent with the sheaths, long acuminate, membranous to slightly coriaceous, without notable lateral keels, apices entire, erose or narrowly bifid, sometimes fimbriate, abaxial surface smooth; **ligules of innovations** 4–15(–20) mm long, slightly to strongly and broadly decurrent with the sheaths, long acuminate, membranous to slightly coriaceous, lateral keels sometimes notable, apices entire or a narrow bifid point, sometimes slightly erose, abaxial surface smooth or sometimes slightly scabrous at the apex. **Leaf blades** (2.5–)5–22 cm long, 0.6–7 mm wide when opened out, flat, conduplicate or involute/convolute and filiform and cylindrical to subelliptical in outline, sometimes opening out to become flat at their apices, straight and erect to slightly curved, glabrous, isomorphic or more or less dimorphic, when dimorphic those of the innovations filiform and cylindrical to subelliptic in outline while those of the upper flowering culm are usually wider and flat, conduplicate or convolute towards the apices, abaxially smooth, adaxially smooth or lightly scaberulous along the veins, sometimes becoming densely scabrous towards the apex, edges smooth or slightly scaberulous, veins usually pronounced, numerous and tightly packed, apex obtuse to slightly pungent; **flag leaf blades** 2.9–6 cm long, 2–7 mm wide when opened out. **Panicles** 10–25 cm long, 3–8 cm wide, open to slightly condensed, oval, greenish-purple with spikelets tending to be laxly glomerate on the distal half of the inflorescence branches with the proximal half usually lacking spikelets, largely exerted to moderately included in the uppermost sheath and/or blade; **main panicle axis** terete to slightly compressed, usually with a narrow groove running down both sides, glabrous, smooth to lightly scaberulous, internodes tending to be long, lower internode 3–11.5 cm long; **panicle branches** 1.5–8 cm long, bearing 10 to over 50 spikelets per branch, flexuous, spreading, pendulous or divergent at a 45° angle to slightly ascending, verticillate in clusters of 2 or 3, terete or slightly grooved, glabrous, almost smooth to scabrous; **pedicels** 0.5–2.5 mm long, usually shorter than the spikelets, glabrous, lightly to densely scabrous. **Spikelets** 1-flowered, sometimes with a rudimentary floret at the apex of the rachilla that appears like a slightly broader section of the rachilla covered in sparse diminute hairs, not strongly laterally compressed, disarticulating above the glumes with the florets disarticulating from the apex of the extended rachilla internode, this remaining attached to the glumes. **Glumes** 3.5–5.5 mm long, subequal, the lower glume 0.2–0.6 mm shorter than the upper glume, lanceolate, membranous, purplish-green, lustrous, smooth or sometimes lightly scabrous throughout the keel of the upper glume; **lower glumes** 1-veined, apex acuminate or bidentate, less frequently finely denticulate or erose; **upper glumes** 3-veined, lateral veins either short < ½ length of glume or reaching from ½ to 2/3 the length of the glume, apex usually acuminate, sometimes finely denticulate. **Floret** stipitate, much shorter than the glumes, never passing the apex of the lower glume. **Lowermost rachilla internode** 0.4–0.7 mm long, prolonged between the glumes and the floret, often slightly geniculate at its apex and bent in a ca. 30°–45° angle, slightly dilated at its apex, usually glabrous, less often with a few long hairs ca. 0.7 mm long emerging from it, smooth. **Lemmas** 2.4–3.5 mm long, of the same consistency as the glumes, light green with purple tinges towards apex, becoming golden at maturity, glabrous, smooth with the keel apex rarely scaberulous, apex truncate and denticulate, usually with 4 clearly distinguished teeth, 0.3–0.5 mm long and erose between the teeth, 5-veined, veins not evident; awns 1.5–4 mm long, sometimes absent, inserted in the middle or lower third of the lemma, usually as long as the lemma or passing the glume apex by as much as 1.5 mm. **Paleas** 0.5–0.8 mm shorter than the lemma, of the same consistency and colour, keels sparsely scabrous and notable, apex bidentate or 4-dentate. **Callus** rounded, short, with a basal tuft of hairs 0.5–2.2 mm long, reaching from 1/3 the length of the lemma to almost the lemma apex. **Rachilla** 1.5–2.5 mm long, reaching from 2/3 to 4/5 the length of the lemma, with copious short to medium-sized hairs 0.5–1.4 mm long, the hairs reaching from 4/5 to sometimes passing the apex of the lemma, apex of rachilla sometimes clavate. **Lodicules** 2, ca. 0.5 mm long, membranous, acute. **Stamens** 3, anthers (0.7–)1.2–1.9 mm long. **Ovary** ca. 0.5 mm long, small, styles 2, stigmas plumose, short. **Caryopses** 1–1.4 mm long, dorsally slightly gibbose, surcus not noticeable, embryo short, hilum basal, oval; **endosperm** dry.

##### Distribution and ecology.

Colombia, Ecuador, Peru, Venezuela. The ecology of this species is distinct compared to many *Calamagrostis* s.l. species as it is usually found in very damp, swamp-like conditions by the side of high-elevation lakes or watercourses in Andean páramo or jalca vegetation, less often in humid open páramo.

##### Other specimens examined.

COLOMBIA. **Boyacá**: Below Las Playas de Ritacuba in the Cocuy mountains above Guican, on the banks of a fast-flowing stream coming off the snow fields and passing through thinly vegetated moraine covered country, 4100 m alt., 24 Jun. 1984, J.R.I. Wood 4457 (K). **Cauca**: Purace National Park, Laguna de San Rafael, in open boggy páramo in the lake basin, particularly in banks by ditches, 3300 m alt., 6 Apr. 1985, J.R.I. Wood 4803 (K); Volcan Purace, above Pilimbala, frequent in bog pools in high páramo, 3700–4000 m alt., 5 Apr. 1985, J.R.I. Wood 4787 (K).

ECUADOR. **Pichincha**: road Olmedo-Laguna San Marcos, W of the pass, 0°5'N; 78°1–2'W, 3600 m alt., 10 Jul. 1980, B. Øllgaard et al. 34406 (K); Along road to Refugio, Volcan Cayembe, páramo and swamp, 00°04'S; 77°54'W, 4300 m alt., 2 Mar. 1988, S. Renvoize 70510 (K). **Napo**: Eastern Cordillera, Llanganati Mountains, by Lake Aucacocha, on stream sides in bog, forming large tussocks up to 20 cm across, 3750 m alt., 16 Aug. 1969, P.J. Edwards 127 (K); Eastern Cordillera, Llanganati Mountains, by Lake Aucacocha, growing in the wettest area of the bog, in clusters of tufts, sheath bases submerged in peat, 3700 m alt., Aug. 1969, P.J. Edwards 62 (K).

##### Notes.

*Deschampsiapodophora* has been traditionally treated as belonging to Calamagrostissubsect.Stylagrostis due to the presence of an extended rachilla internode between the glumes and the floret. *Deschampsiapodophora* is closely related to *D.parodiana* (=*Calamagrostisligulata*) and has been placed as a synonym of this in previous works (Escalona 1988a, 1988b; Tovar 1993; Luteyn 1999; Bono 2010; Briceño 2010). The principal differentiating characters that separate *Deschampsiaparodiana* from *D.podophora* are the smaller anthers, 0.4–0.5 mm long (vs. (0.7–)1.2–1.5(–1.9) mm long in *D.podophora*) and the shorter rachilla, 1–1.2 mm long, that is sparsely pilose with hairs not usually reaching the apex of the palea (vs. (1.2–)1.4–2.5 mm long, with copious hairs that usually surpass the lemma in *D.podophora*). Characters of shape and density of the inflorescence mentioned in Laegaard (1998: 27) were found to be not good for differentiating the two species. While *D.parodiana* only has a lax open inflorescence with long pendant branches and spikelets glomerate on the distal part of the branches, *D.podophora* exhibits both denser semi-spikelike inflorescences with inflorescence branches having spikelets from the base as well as open inflorescences with pendulous branches and spikelets glomerate on the distal part of the branches like that of *D.parodiana*.

Some specimens from Colombia were found to be generally larger than those from Ecuador, in terms of the number and length of the culms with mainly cauline leaf blades that were longer and wider. These specimens had a habit appearance similar to var. mutica being larger tussock-forming plants with multiple culms and inflorescences held within sheaths.

#### Deschampsia podophora (Pilg.) Saarela var. mutica

Taxon classificationPlantaePoalesPoaceae

Sylvester var. nov.

urn:lsid:ipni.org:names:60478842-2

Fig. 3

##### Type.

COLOMBIA. Cundinamarca: Mun. Santa Rosa-Usme, Sumapaz páramo, by Laguna Larga, in swamp at the edge of the lake in open páramo country, 3700 m alt., 19 Aug. 1985, J.R.I. Wood 5033 (holotype: FMB (FMB11918!); isotypes: COL (COL000434793!), K [2 sheets]!).

**Figure 3.**
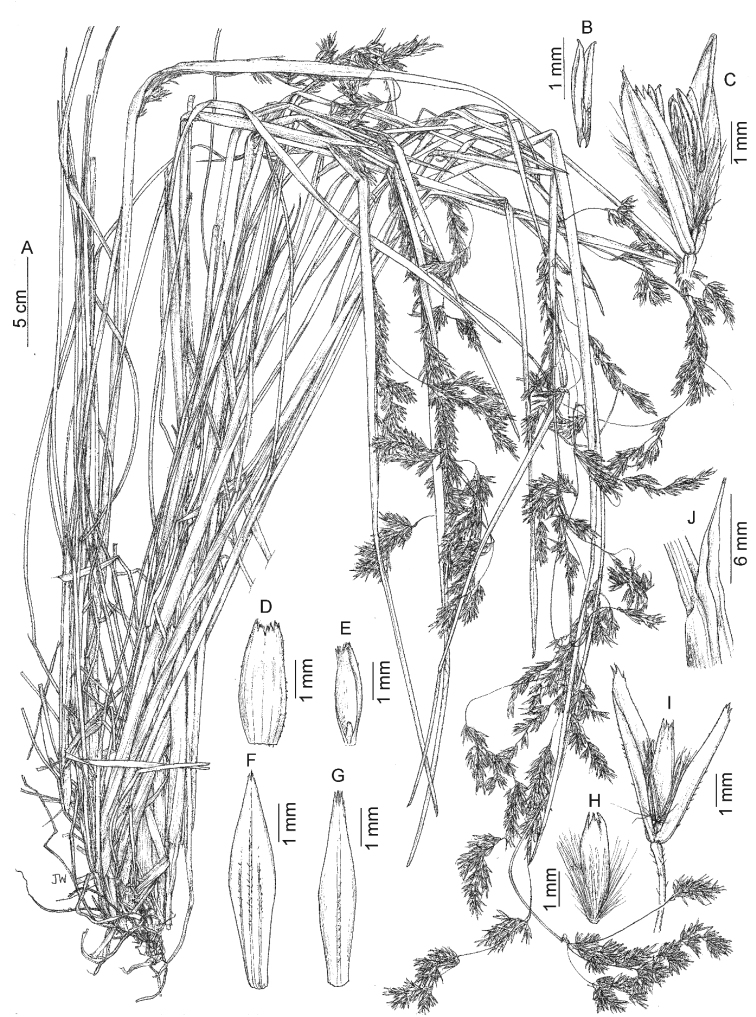
Deschampsiapodophoravar.mutica. **A** whole plant **B** anther **C** spikelet at maturity with anthers **D** lemma, abaxial view **E** palea, abaxial view **F** upper glume, abaxial view **G** lower glume, abaxial view **H** floret **I** spikelet and pedicel, lateral view **J** ligule; drawn by Juliet Beentje from the isotype, J.R.I. Wood 5033 (K).

##### Diagnosis.

Deschampsiapodophoravar.mutica differs from *D.podophora* by the lemmas being muticous and lacking awns (vs. lemmas usually with a well-developed dorsal awn inserted in the lower or middle third of the spikelet, measuring 1.5–3.5(–4) mm long and usually not surpassing the glumes), tussock-forming habit with multiple culms and leaves mainly cauline, without a basal mat clearly shorter than the flowering culms (vs. plants forming short isolated tufts with solitary culms and the leaves forming a short basal mat clearly shorter than the largely exerted flowering culms), inflorescences often sub-included in the sheaths and blades (vs. flowering culms largely exerted from basal mats); leaf blades 11–22 cm long, 0.35–0.6 mm wide when rolled, often dimorphic, those of the innovations filiform and cylindrical to subelliptic in outline, while upper flowering culm blades 6.5–25 cm long, 2–7 mm wide when opened out, usually wider and flat, conduplicate or convolute towards the pungent apices (vs. leaf blades 5–8 cm long, 2–3 mm wide, not clearly dimorphic, with all blades flat or conduplicate, apices obtuse), ligules 4–11 mm long (vs. ligules generally longer, 10–22 mm long), anthers (1.4–)1.8–1.9 mm long (vs. anthers (0.7–)1.2–1.5 mm long), upper glume lateral veins reaching from ½ to 2/3 the length of the glume (vs. upper glume lateral veins short, < ½ length of glume).

##### Description.

**Tufted perennial** with vertical rhizomes forming medium-sized tussocks with leaf blades both basal and cauline and some cauline blades often surpassing the inflorescence. **Culms** 26–110 cm tall, to 3 mm wide, erect, striate, nodes and internodes terete, smooth and lustrous; nodes hidden in the sheaths with no nodes exposed at flowering; uppermost internodes 23–32.5 cm long, not, or not pronouncedly, longer than the sheath; **Sheaths** striate; **flag leaf sheaths** 22–38 cm long; **upper culm sheaths** lax, glabrous and smooth; **basal leaf sheaths** 4.5–20 cm long, glabrous and smooth, longer than the internodes. **Ligules** not stipulate; **upper culm ligules** 7.5–11 mm long, strongly decurrent with the sheaths, long acuminate, membranous to slightly coriaceous, without notable lateral keels, apices erose or narrowly bifid, sometimes fimbriate, abaxial surface smooth; **ligules of innovations** 4–9 mm long, slightly to strongly decurrent with the sheaths, long acuminate, membranous to slightly coriaceous, lateral keels sometimes notable, apices a narrow bifid point, sometimes slightly erose, abaxial surface smooth or sometimes slightly scabrous at the apex. **Leaf blades** sometimes dimorphic, those of the innovations filiform and cylindrical to subelliptic in outline while those of the upper flowering culm are usually wider and flat, conduplicate, or convolute towards the apices; **leaf blades of innovations** 11–22 cm long, 0.35–1 mm wide when rolled or folded, narrow and conduplicate or filiform involute or convolute and cylindrical to subelliptical in outline, rarely completely flat, sometimes opening out to become flat at their apices, straight and erect, glabrous, abaxially smooth, adaxially lightly scaberulous along the veins or rarely smooth, edges smooth or slightly scaberulous, apex obtuse to slightly pungent; **leaf blades of lower flowering culm** to 1.2 mm wide when rolled or folded, similar to those of the innovations or slightly wider; **leaf blades of upper flowering culm** 6.5–25 cm long, 2–7 mm wide when opened out, flat, conduplicate or convolute towards the apices, glabrous, abaxially smooth to finely scaberulous, sometimes becoming densely scabrous at the apex, adaxially smooth or scaberulous towards the margins, veins pronounced, numerous and tightly packed, edges smooth or slightly scaberulous, apex acute to pungent; **flag leaf blade** ca. 2.9 cm long, recurved, slightly narrower than the basal blade. **Panicles** 10–25 cm long, 5–8 cm wide, open and diffuse with main axis having long internodes, oval, usually slightly to moderately included in the uppermost sheath and/or blade, greenish-purple, spikelets tending to be laxly glomerate on the distal half of the inflorescence branches with the proximal half usually lacking spikelets; **main panicle axis** terete to slightly compressed, usually with a narrow groove running down both sides, glabrous, smooth to lightly scaberulous, internodes tending to be very long, lower internode 5.5–11.5 cm long; **panicle branches** flexuous, spreading, pendulous or divergent at a 45° angle to slightly ascending; primary panicle branches 1.5–8 cm long, bearing 10 to over 50 spikelets per branch, terete and slightly grooved, verticillate in clusters of 2 or 3, glabrous, almost smooth to lightly scabrous; **pedicels** 0.5–2.5 mm long, usually shorter than the spikelets, glabrous, lightly scabrous. **Glumes** 4.5–4.9 mm long, subequal, the lower glume 0.3–0.6 mm shorter than the upper glume, membranous, purplish-green, lustrous, smooth or sometimes lightly scabrous throughout the keel of the upper glume; **lower glume** 1-veined, apex usually bidentate, less frequently finely denticulate or erose; **upper glume** 3-veined, lateral veins reaching from ½ to 2/3 the length of the glume, apex usually acuminate, sometimes finely denticulate. **Floret** stipitate, much shorter than the glumes, never passing the apex of the lower glume. **Lemmas** 2.8–3.4 mm long, of the same consistency as the glumes, light green with purple tinges towards apex, becoming golden at maturity, glabrous, smooth with the keel apex rarely scaberulous, apex truncate and denticulate, usually with 5 clearly distinguished teeth, 0.3 mm long, and erose between the teeth, 5-veined, veins not evident; muticous and lacking an awn. **Rachilla** 1.5–2.5 mm long, reaching from 2/3 to 4/5 the length of the lemma, with copious short to medium-sized hairs 0.5–1.4 mm long, the hairs reaching from 4/5 to almost the apex of the lemma and usually surpassing the palea, apex of rachilla often clavate. **Stamens** 3, anthers (1.4–)1.8–1.9 mm long.

##### Distribution and ecology.

Endemic to Colombia. Known from páramos of the Cordillera Oriental and Cordillera Central of the Colombian Andes. For the Cordillera Oriental, the species is known from Departamento Cundinamarca municipalities Usme and Santa Rosa and Páramo Pisba of Departamento Boyacá. For the Cordillera Central, the species is known from Páramo del Quindio and Páramos de la Laguna del Mosquito of Departamento Caldas. Found in humid, swampy areas, often by rivers or lakes and less often in more mesic habitats, such as road verges (presumed damp). The type specimens were collected from swampy areas bordering the Laguna Larga of the Sumapaz páramo in Cundinamarca. The holotype shows signs of grazing, with blades and culms abruptly cut.

##### Other specimens examined.

COLOMBIA. **Boyacá**: Mun. Socota: Páramo Pisba, Peña Negra, lagoon “Choro Negro”, páramo with *Calamagrostiseffusa*, *Espeletia* sp., *Chusquea* sp., *Diplostephium* sp. etc., swampy, 3500 m alt., 11 Feb. 1999, D. Stančik & S. Medina 2351 (COL-000184768; FMB-051200); Páramo Pisba, Alto de Calarca, humid grassy páramo with *Calamagrostiseffusa*, *Espeletia* sp., *Chusquea* sp. et bunch Grass, 3600 m alt., 11 Feb. 1999, D. Stančik & S. Medina 2331 (FMB-046617). **Caldas**: Páramo del Quindio, swale in páramo valley, 3700–4200 m alt., 15–20 Aug. 1922, F.W. Pennell & T.E. Hazen 9949 (K); Cordillera Central, cabeceras del río Otúm, bajando del Nevado de Santa Isabel, páramos de la Laguna del Mosquito, 3820 m alt., 26 Nov. 1946, J. Cuatrecasas 23233 (K). **Cundinamarca**: Mun. Usme: Laguna Chizaca, hierba creciendo al lado de la carretera que conduce a la laguna, 26 Jul. 1986, A. Betancur & M. Palacio 44 (HUA-47083); Páramo de Chisaca, growing in marsh near lagoon, 3750 m alt., 30 Sep. 1966, T.R. Soderstrom 1273 (K).

##### Preliminary conservation status.

Vulnerable (VU). Despite the species being known from several collections from the Central and Eastern Cordilleras of the Colombian Andes, the páramos of Colombia are currently facing threats of habitat degradation and loss, principally from mining (Pérez-Escobar et al. 2018) and so a preliminary conservation status of VU is given.

##### Etymology.

The varietal epithet refers to the absence of an awn inserted in the dorsal surface of the lemma.

##### Notes.

Certain specimens were encountered which exhibited a mix of the characters mentioned in the diagnosis for separating *D.podophora* from D.podophoravar.mutica. For example, specimen Cuatrecasas 23233 from the Cordillera Central of Colombia had isomorphic flat leaf blades which formed a basal mat much shorter than the exerted culms but also had awnless spikelets. A few other specimens exhibited the larger habit, i.e. multiple longer culms with longer and wider leaf blades forming tussocks and inflorescences included within sheaths, but with awned spikelets.

Specimens of Deschampsiapodophoravar.mutica were commonly misidentified as *Poa* L. in herbaria, most likely due to the lemmas lacking awns. There are very few South American *Calamagrostis* s.l. that consistently lack awns (see ‘notes’ of *Deschampsiasantamartensis* sp. nov. above) and, of the species not belonging to subsect. Stylagrostis (=*Deschampsia*), the closest resembling species is *Calamagrostisecuadoriensis*Laegaard (1998), which also has short florets with a pilose rachilla extension and which lack awns. However, *C.ecuadoriensis* can be differentiated from Deschampsiapodophoravar.mutica by, amongst other things, a) habit, being small tussocks, 20–30 cm high, with leaves mostly basal; b) culms, panicle branches and pedicels densely hispid; c) panicles narrow, ca.1 cm wide; d) anthers 0.8–1 mm long; e) florets only shortly stipitate, with the stipe < 0.15 mm long at the base of the glumes.

### Five new records of *Calamagrostis* for Colombia

#### Calamagrostis cf. carchiensis

Taxon classificationPlantaePoalesPoaceae

Lægaard, Novon 8(1): 23–25, f. 1A. 1998.

Fig. 4

##### Type.

ECUADOR. Napo [Sucumbíos]: Páramo de Mirador above Cocha Seca, lower páramo zone, burned, 00°34'N; 77°39'W, 3700–3900 m alt., 23 May 1985, S. Lægaard 54413 (holotype: AAU!; isotypes: K (K000308461!), MO (MO05100301 [image!]), QCA (QCA78857 [image!]), QCNE, US (US00588939!)).

**Figure 4.**
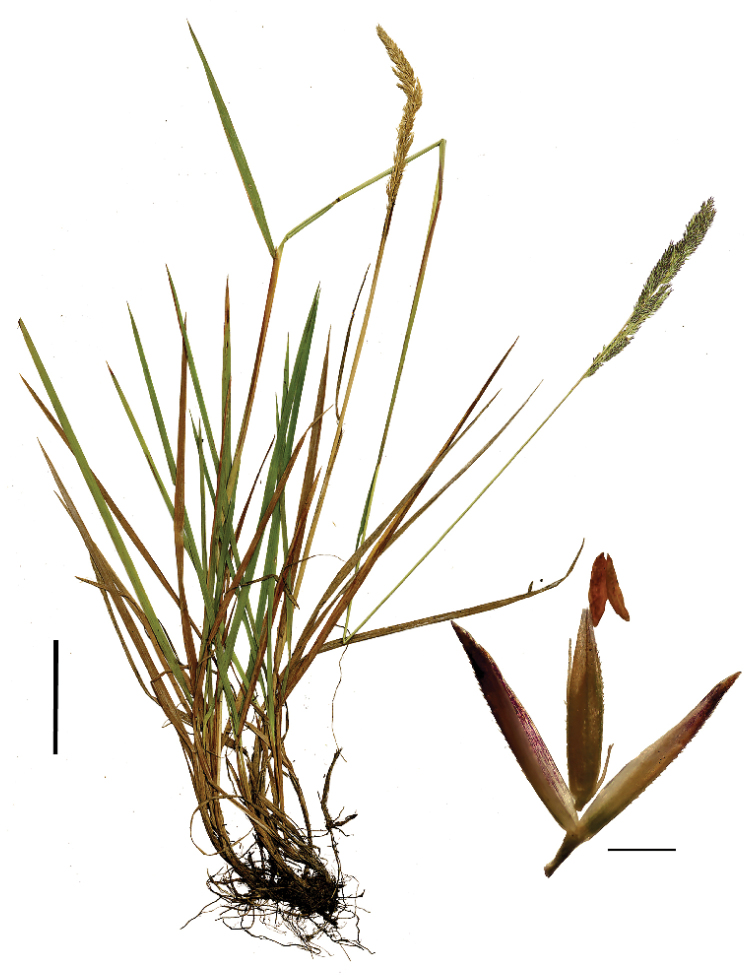
Calamagrostiscf.carchiensis Lægaard general habit (scale bar 5 cm) and spikelet. Scale bar: 1 mm; S.P. Sylvester 3049 (FMB).

##### Comments.

Previously considered endemic to Ecuador with a global conservation status of VU B1ab(iii) - Vulnerable (León Yánez et al. 2011). The voucher specimen collected matches the species description in every aspect apart from its having two anthers as opposed to one. The number of anthers is taxonomically informative in the genus *Calamagrostis* and it may be that the Sylvester 3049 specimen should be considered as a distinct taxon, although further research including molecular analysis is needed to clarify this. Laegaard (1998) noted that *Calamagrostiscarchiensis* bears affinity to *Calamagrostisbogotensis* (Pilg.) Pilg., especially in terms of florets with a single anther.

##### Specimens examined.

COLOMBIA. **Boyacá**: Mun. Duitama, páramo de Agueros, via que conduce a Vereda de Avendanos, 05°54.527'N; 73°03.761'W, 3445 m alt., 28 Oct. 2017, S. P. Sylvester, W. Bravo & J. Aguilar 3049 (COL, FMB, K, US).

#### Calamagrostis guamanensis

Taxon classificationPlantaePoalesPoaceae

Escalona, Phytologia 65(5): 340, f. 2. 1988.

Fig. 5 A, B

##### Type.

ECUADOR. Napo: [road Quito-Baeza at the telecomunication antenna, N of the Guamani paramo, in the oriental Andes, 0°10.2'S; 78°23.4'W], 4260 m alt., 3 Mar. 1985, [grass forming loose tufts in cushion plants of *Distichiamuscoides*], F.D. Escalona & Gallegos 390 (holotype: ISC; isotypes: K!, MO (MO115961), QCA, US!, VEN).

##### Comments.

Previously considered endemic to Ecuador (Luteyn 1999).

##### Specimen examined.

COLOMBIA. **Nariño**: Mun. Pasto, Volcan Galeras, frequent growing in cushion plants and similar damp protected places at high altitudes in base high páramo, 1°13.6417'N; 77°21.8718'W, 4000 m alt., 29 Nov. 1983, J.R.I Wood 4064 (FMB, K).

**Figure 5.**
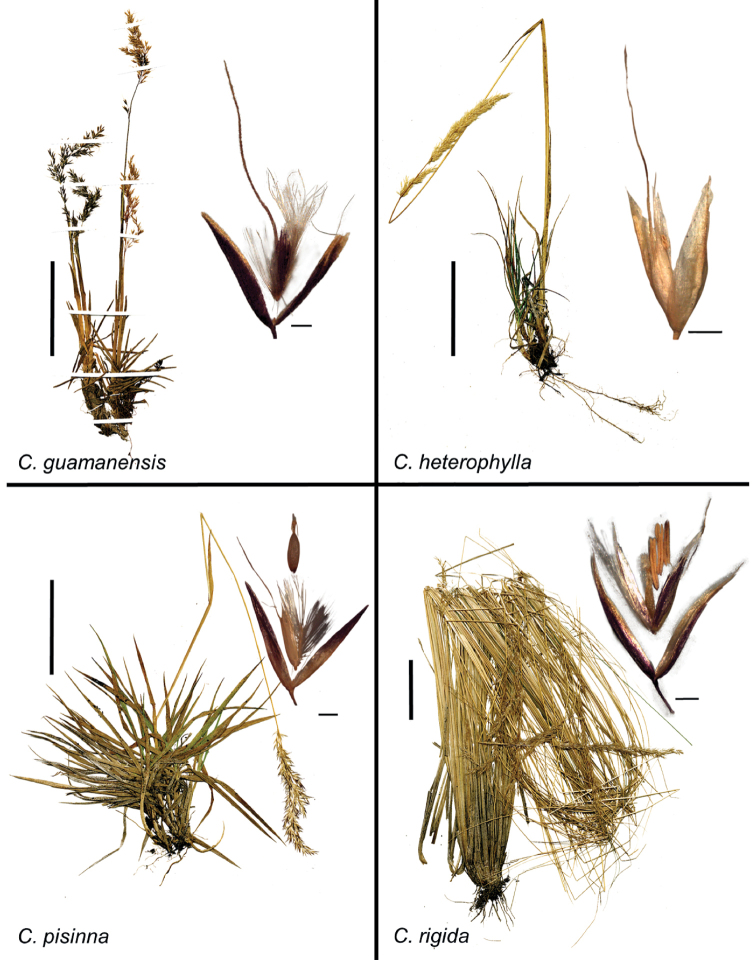
A general habit and a spikelet of *Calamagrostisguamanensis* Escalona, J.R.I. Wood 4064 (FMB), *Calamagrostisheterophylla* (Wedd.) Pilg., S.P. Sylvester 3158 (US), *Calamagrostispisinna* Swallen, S.P. Sylvester 3107 (US), and *Calamagrostisrigida* (Kunth) Trin. ex Steud., S.P. Sylvester 3125 (US). Habit scale bar 5 cm; spikelet scale bar 1 mm.

#### Calamagrostis heterophylla

Taxon classificationPlantaePoalesPoaceae

(Wedd.) Pilg., Bot. Jahrb. Syst. 42: 64. 1908. Deyeuxia heterophylla Wedd., Bull. Soc. Bot. France 22: 177, 180. 1875.

Fig. 5 C, D

**Type.** Bolivia. [Potosí], A. D’Orbigny 202 (lectotype, designated by Rúgolo de Agrasar (2012: 201): P (P00729766 [image!]); isolectotypes: BAA (BAA00001845 [image!]), US (US00133531!), W (W18890120011 [image!])).
= Chaetotropisandina Ball, J. Linn. Soc. Bot. 22: 58. 1885. Type: Peru. Ex Saxosis Andium Peruviae juxta Paquim Chicla, 12000–13000’ s.m. [3658–3962 m alt.], 21–23 Apr. 1882, J. Ball s.n. (holotype: P; isotypes: BAA (BAA00001704 [image!]) fragm., K, US (US00344798!) fragm. ex K & ex LE).
= Calamagrostisheterophylla(Wedd.)Pilg.var.robustior Pilg., Bot. Jahrb. Syst.: 64. l908. Type: Peru. Puno: Azangaro, in saxosis calcareis, 4000 m alt., Feb. 1902, A. Weberbauer 474 (holotype: B (not found); isotype: US (US00153711!)).
= Calamagrostisheterophylla(Wedd.)Pilg.var.pubescens Pilg., Bot. Jahrb. Syst.: 64. 1908. Type: Peru. Puno: in provincia Sandia, supra Cuyocuyo, in campis fructisibus nonnullis intermistix, 3700–3800 m alt., May 1902, A. Weberbauer 905 (holotype: B (not found); isotype: US (US00153712!)).
= Calamagrostiscalvescens Pilg., Bot. Jahrb. Syst. 42: 65. 1908. Type: Peru. Ancash: Prov. Cajatambo, 3000-3300 m alt., 13 Apr. 1902, A. Weberbauer 2842 (lectotype, designated by Nicora and Rúgolo de Agrasar (1998: 168): BAA (BAA00000758 [image!]) fragm. ex B; isolectotypes: MOL, US (US00131526!)).
= Calamagrostismulleri Luces, Bol. Soc. Venez., Cienc. Nat. 15 (80):9. 1953. Type: Venezuela. Edo. Mérida: Páramo de Mucuchíes, 4000 m alt., 11 Nov. 1939, A.S. Müller 897 (holotype: VEN (VEN20682 [image!]); isotypes: MO, US (US00149289!)).
= Calamagrostismacbridei Tovar, Mem. Mus. Javier Prado 11:63. 1960. Type: Perú. Pasco, Huarón, northern part Cerro Pasco, northeastern slope, 14000 ft. [4267 m] alt., 12 Jun. 1922, J.F. Macbride & W. Featherstone 998 (holotype: US (US00153707!); isotypes: F (F0040679F [image!]), SI (SI000619 [image!]) fragm.).
= Calamagrostisswallenii Tovar, Mem. Mus. Hist. Nat. “Javier Prado” 11: 66. 1960. Deyeuxiaswallenii (Tovar) Rúgolo, Rev. ~Deyeuxia~ Bolivien 128. 1995. Type: Peru. Huancavelica: Prov. Huancavelica: Tausiri, cerca a Manta, pajonal de Puna, 4500 m alt., 31 Mar. 1953, O. Tovar 1168 (holotype: US (US00133195!); isotypes: GH (GH00023323 [image!]), K (K000308446!), MO (MO115821 [image!]), USM (USM000722 [image!])).

##### Comments.

Previously known from high Andean regions of Venezuela (Hokche et al. 2008; Bono 2010; Briceño 2010), Peru (Tovar 1993), Bolivia (Villavicencio 1995, 1998), northern Chile and northwest Argentina (Rúgolo de Agrasar 2012). The specimens collected exhibit characters of both *Calamagrostisheterophylla* and *Calamagrostisbrevipaleata* Swallen, an Ecuadorian endemic, with both species having heteromorphic leaf blades, the cauline glabrous and those of the innovations pilose. The species bear more affinity to *C.heterophylla* as the lemmas measure less than 4.2 mm (> 5 mm long in *C.brevipaleata*) and the leaf blades measure < 10 cm long (10–25 cm long in *C.brevipaleata*). However, the specimens do have characteristics of *C.brevipaleata* in terms of the lemma surfaces, which are smooth proximally and scabrous distally and no great differentiation in width between the cauline and tiller leaf blades.

##### Specimen examined.

COLOMBIA. **Boyacá**: Mun. Chiscas, páramo El Penon, borde de bosque de *Polylepis* creciendo sobre roca, 6°36.0714'N; 72°26.229'W, 3917 m alt., 5 Mar. 2018, S.P. Sylvester, R.J. Soreng, W. Bravo & L.E. Cuta 3158 (COL, FMB, K, US).

#### Calamagrostis pisinna

Taxon classificationPlantaePoalesPoaceae

Swallen, Contr. U.S. Natl. Herb. 29(6): 257–258. 1948[1949].

Fig. 5E, F

##### Type.

Venezuela. Mérida: rocky ridges, higher paramos, near El Gavilon, 4200 m alt., 25 Jan. 1929, H. Pittier 13277-1/2 (**lectotype, designated here**: US (US00149283! [A-two flowering culms are the type, B-on left side of sheet is unknown])).

##### Comments.

Previously considered endemic to Venezuela (Escalona 1988a) but specimens have been found in the Sierra Nevada del Cocuy of the Cordillera Oriental of Colombia. Specimen *Sylvester 3107* differed slightly from the species described from Venezuela in that the leaf blades were densely pilose and often found to be conduplicate (Fig. 5E, F). As only one flowering specimen was encountered of this morphotype and specimens being found growing out of fairly inaccessible crag ledges, more collections need to be made to ascertain whether this may be a distinct species. All specimens from the Sierra Nevada del Cocuy have a rachilla extension with hairs that reach or surpass the apex of the floret, while Venezuelan specimens have rachilla hairs which usually do not surpass the apex of the palea (Escalona 1988a). Saarela et al. (2017) found one of the specimens of *C.pisinna* that we cite here (*Cleef 8653*) to have an unusual placement in plastid analyses (no nuclear ribosomal data was obtained) as a basally diverging lineage in a moderately to strongly supported clade that also contained *Lagurusovatus* L., Aveninae s.str., and Koeleriinae excluding *L.ovatus*. However, there is a phylogenetic discrepancy between matK and PsbK sequences reported by Saarela et al. (2017) for *C.pisinna* that needs to be evaluated before any taxonomic conclusions can be drawn.

There is a sterile tuft on the left side of the Pittier type sheet that is a different species and quite possibly a different genus, having acicular involute blades and longer acute ligules that are decurrent (p.p. b). Thus, we lectotypify to the two flowering tufts on the right side of the type sheet (p.p. a), which are identical and fit the description in the type protologue.

##### Specimens examined.

COLOMBIA. **Boyacá**: Mun. Chiscas, páramo de Chacaritas, limit between páramo and superpáramo, growing out of rock ledge, 6°37.6794'N; 72°23.6616'W, 4072 m alt., 4 Mar. 2018, S.P. Sylvester, R.J. Soreng, W. Bravo & L.E. Cuta 3107 (US); Sierra Nevada del Cocuy, Páramo Cóncavo, Cueva de los Hombres, 3 km N del Morro Púlpito del Diablo, 4350 m alt., 28 Feb. 1973, A. Cleef 8610 (US-01234789); 4350 m alt., A. Cleef 8653 (US-01234736).

#### Calamagrostis rigida

Taxon classificationPlantaePoalesPoaceae

(Kunth) Trin. ex Steud., Nomencl. Bot. (ed. 2) 1: 251. 1840. Deyeuxia rigida Kunth, Nov. Gen. Sp. (quarto ed.) 1: 144. 1815[1816]. Arundo rigida (Kunth) Poir., Encycl. 4: 705. 1816.

Fig. 5 G, H

**Type.** ECUADOR. In planitie frigida Antisanae [inter speluncam, Machay de Antisana], et Chussulongo, 2200 hexap. (4023 m alt.)], Regni Quitensi [Pichincha], 1833, A.J.A. Bonpland 2271 [s.n.] (holotype: P (P00729827 [image!]); isotypes: BM (BM000938560 [image!]), GH (GH00023418 [image!]) fragm., LE (LE-TRIN-1804.01!) fragm. ex Herb. Humb., P (P00129585 [image!], P00740362 [image!], P026296 [image!]), US (US00406356!) fragm., W???).
= Deyeuxiagracilis Wedd., Bull. Soc. Bot. Fr. 22: 179. 1875. Calamagrostisgracilis (Wedd.) Pilg., Bot. Jahrb. Syst. 42: 71. 1908. Calamagrostisgracilis (Wedd.) Henrard, Mede. Rijks-Herb. 40: 61. 1921, nom. illeg. hom. Type: BOLIVIA. Province de Larecaja, Cordillera de Sorata, 1851 [1857], Weddell s.n. (holotype: P (P00729786 [image!]); isotypes: P (P030111 [image!]), S (S-R-1458 [image!]), US (US00479086!, US00479087!)).
= Deyeuxiasulcata Wedd., Bull. Soc. Bot. Fr. 22: 178–180. 1875. Type: BOLIVIA. Prov. Larecaja, viciniis Sorata [Andes de Sorata], Puerta del Inca, prope trincheras Chiliata, in Scopulosos, Reg. Alp., 3800 m alt., Mar. [Apr.] 1858 [1868], G. Mandon 1308 bis. (holotype P; isotypes: GH (GH00023431 [image!]), JE (JE00014185 [image!]) fragm., L (L0044085 [image!], L0044083 [image!]), NY (NY380547 [image!]), P (P030105 [image!]), S (S-R-1469 [image!]), US (US00149252!) fragm.).
= Calamagrostisantoniana Steud. ex Lechler, Berberid. Amer. Austr. 56. 1857, nom. nud.
= Agrostisantoniana Griseb., Abh. Königl. Ges. Wiss. Göttingen 24: 293. 1879. Calamagrostisantoniana (Griseb.) Hack. ex Dusén, Rep. Princeton Univ. Exp. Patagonia, Botany, Suppl. 8: 42. 1915. Calamagrostisantoniana (Griseb.) Steud. ex Hitchc., Contr. U.S. Natl. Herb. 24(8): 378. 1927. Deyeuxiaantoniana (Griseb.) Parodi, Revista Argent. Agron. 20: 14. 1953. Calamagrostisantoniana (Griseb.) D.M. Moore, Fl. Tierra del Fuego 310. 1983, comb. illeg. hom. Type: ARGENTINA: Salta: Umgebung des Nevado del Castillo [Alrededores del Nevado del Castillo], 10–13000 ft [3048–3962 m alt.], 19–23 Mar. 1873, P.G. Lorentz & G.H.E.W. Hieronymus 67 + 72 (lectotype, designated here: GOET (GOET006107 [image!]); isolectotypes: GOET (GOET006106 [image!], GOET006108 [image!]); P.G. Lorentz & G.H.E.W. Hieronymus 67 isolectotypes: BAA (BAA00001327 [image!]) fragm., CORD (CORD00004685 [image!], CORD00004686 [image!]); P.G. Lorentz & G.H.E.W. Hieronymus 72 isolectotypes: BAA (BAA00001326 [image!]) fragm., CORD (CORD00004687 [image!], CORD00004688 [image!], CORD00004689 [image!]), K [cited by Rúgolo de Agrasar 2012: 208, but not seen], US (US00406312!, US00406404!)). Other original material: BOLIVIA [PERU]. Viciniis Sorata, prope Milipaya, in scopulosis, reg. alpina, 3700 m alt., Mar.–May 1861, G. Mandon 1308 (GOET (GOET006230 [image!])). PERU. In graminosis pr. San Antonio rara, Jun. [1854], [W. Lechler s.n.], W. Lechler Pl. Peruv. 1800 (BAA (BAA00001648 [image!]) fragm., BR (BR0000006865757 [image!]), G (G00099542 [image!], G00099543 [image!]), GOET (GOET006109 [image!]), K (K000308438 [image!]), LE (LE00009360 [image!], LE00009361 [image!]), M?, P (P00729795 [image!], P00729844 [image!], P00740472 [image!]), S (S-R-7637 [image!]), TUB (TUB009259 [image!]), US (US00131135!), W (W1889-0241764 [image!])).
= Calamagrostissandiensis Pilg., Bot. Jahrb. Syst. 42: 68. 1908. Type: PERU. Prope Cuyocuyo, provincia Sandia, 3700–3800 m alt., 3 May 1902, Weberbauer 906 (lectotype, designated by Vega and Rúgolo de Agrasar (2013: 28): MOL; isolectotypes: BAA (BAA00000768 [image!] fragm. ex B, S (S-R-834 [image!]), US (US00149279!) fragm.).
= Calamagrostisgusindei Pilg. ex Skottsberg, Acta Horti Gothob. 2: 29. 1926. Deyeuxiagusindei (Pilg ex Skottsb.) Parodi, Revista Argent. Agron. 20: 14. 1953. Type: CHILE. Feuerland, Beagle [Beagle Kanal, zwischen (between) Steinen am Ufer; Tierra del Fuego, Remolino, Canal de Beagle], Mar. 1923, P. Gusinde 40 (holotype: BG; isotypes: BAA (BAA00000761 [image!]) fragm., W (W1941-0001573 [image!])).
= Deyeuxiacrassifolia Hack. ex Sodiro, Revista Col. Nac. Vicente Rocafuerte 12: 64, 73. 1930. Type: ECUADOR. Crece en los pajonales del Monte Pichincha, Sep. [Jun.] 1887 [1886], Sodiro 25/9 [s.n.] (holotype: Q; isotypes: QPSL, S (S-R-1457 [image!]), US (US00406342!), W (W1916-0038065 [image!], W1916-0038066 [image!])).

##### Comments.

Previously considered to have its northernmost distribution in Ecuador (Rúgolo de Agrasar 2012), although Luteyn (1999) mentions its presence in Costa Rica, this is the first record of *C.rigida* for Colombia. It has been erroneously determined as *Calamagrostisrecta* (Kunth) Trin. ex Steud. The latter differs from *C.rigida* by the rachilla hairs reaching up to ¾ the length of the lemma (as opposed to usually reaching to slightly surpassing the lemma apex in *C.rigida*), the awns being generally longer (6.2–7.5 mm as opposed to 4–6 mm in *C.rigida*) and only slightly surpassing the glumes, the lemma apex being slightly bidentate (as opposed to bifid in *C.rigida*) and the ligule being generally truncate and shorter, 1–5(–6.6) mm long (as opposed to long acuminate, (3–)8–12 mm long in *C.rigida*), amongst other things.

There has been discrepancy regarding the typification of *Agrostisantoniana*, with previous research (e.g. Rúgolo de Agrasar 2012) citing the specimen P.G. Lorentz and G.H.E.W. Hieronymus 72 housed at CORD herbarium from Argentina as holotype, while the protologue mentions this (although not explicitly giving collector and number) as well as three other collections: Spruce pl. ecuad. 5927 presumably from Ecuador, G. Mandon 1308 from Bolivia [Peru] and W. Lechler 1800 from Peru. A further specimen, P.G. Lorentz and G.H.E.W. Hieronymus 67, makes a total of at least five syntypes for this name, although the three GOET Lorentz and Hieronymus syntypes were annotated by Grisebach with “67 + 72”. Hitchcock (1927) gave a partial lectotypification by dictating the type as Lechler 1800, but did not indicate the herbarium. The Lechler 1800 specimen was distributed with the following label annotated by Hohenacker: “W. Lechler Pl. Peruvian. Ed. R. F. Hohenacker 1800 Calamagrostis Antoniana Steud. Ipse [“he himself said it”]. In graminosis pr. San Antonio rara Jun. m.”. As no annotations by Grisebach were found on any of the Lechler 1800, Mandon 1308 or Lorentz and Hieronymus 67 and 72 CORD specimens (Spruce pl. ecuad. 5927 specimens not found), with only Lechler 1800 specimens at P being verified by Steudel, we lectotypify to the best of the three GOET Lorentz and Hieronymus 67 + 72 syntypes annotated by Grisebach and consider syntypes Lorentz and Hieronymus 67 and Lorentz and Hieronymus 72 to be isolectotypes.

##### Specimens Examined.

COLOMBIA. **Boyacá**: Mun. Chiscas, páramo de Chacaritas, asociado a rocas de 4 m de altura, 6°37.3362'N; 72°23.424'W, 4192 m alt., 4 Mar. 2018, S.P. Sylvester, R.J. Soreng, W. Bravo & L.E. Cuta 3119 (COL, FMB, US, UPTC); Mun. Chiscas, páramo de Chacaritas, arribando a la morrena, 6°37.0674'N; 72°23.3394'W, 4354 m alt., 4 Mar. 2018, S.P. Sylvester, R.J. Soreng, W. Bravo & L.E. Cuta 3125 (COL, FMB, K, SI, UPTC, US); Mun. Chiscas, páramo de Chacaritas, arribando a la morrena, 6°37.0674'N; 72°23.3394'W, 4354 m alt., 4 Mar. 2018, S.P. Sylvester, R.J. Soreng, W. Bravo & L.E. Cuta 3126 (US). Dep. Santander: Páramo de la Angostura, Vereda El Mortino, Ubicada en borde de quebrada, 6°57.5'N; 72°43.5'W, 3605 m alt., 17 Nov. 2007, M.C. Gomez 1 (US).

## Supplementary Material

XML Treatment for Calamagrostis meridensis

XML Treatment for Deyeuxia sodiroana

XML Treatment for Calamagrostis

XML Treatment for Calamagrostis spruceana

XML Treatment for Deyeuxia pendula

XML Treatment for Calamagrostis planifolia

XML Treatment for Calamagrostis macrophylla

XML Treatment for Calamagrostis crispifolius

XML Treatment for Deschampsia santamartensis

XML Treatment for Deschampsia podophora

XML Treatment for Deschampsia podophora (Pilg.) Saarela var. mutica

XML Treatment for Calamagrostis cf. carchiensis

XML Treatment for Calamagrostis guamanensis

XML Treatment for Calamagrostis heterophylla

XML Treatment for Calamagrostis pisinna

XML Treatment for Calamagrostis rigida

## Supplementary materials

Supplementary material 1 Supplementary key (in spanish) Data type: species data Explanation note: Spanish version of identification key to the 54 species of *Calamagrostis* s.l. known or likely to occur in the páramos of north-west South America and southern Central America. This dataset is made available under the Open Database License (http://opendatacommons.org/licenses/odbl/1.0/). The Open Database License (ODbL) is a license agreement intended to allow users to freely share, modify, and use this Dataset while maintaining this same freedom for others, provided that the original source and author(s) are credited.Steven P. Sylvester, Robert J. Soreng, William J. Bravo-Pedraza, Lia E. Cuta-Alarcon, Diego Giraldo-Cañas, Jose Aguilar-Cano, Paul M. Peterson
