# Supplementary material for: Páramo Calamagrostis s.l. (Poaceae): An updated list and key to the species known or likely to occur in páramos of NW South America and southern Central America including two new species, one new variety and five new records for Colombia
Source: PhytoKeys. 2019 May 28;122:29–78. doi: 10.3897/phytokeys.122.33032 (PMC6548746; doi:10.3897/phytokeys.122.33032)
Supplement: Supplementary material 1 [file phytokeys-122-029-s001.pdf]

SUPPLEMENTARY KEY (IN SPANISH): CLAVE A LAS ESPECIES DE *CALAMAGROSTIS* S.L. CONOCIDAS O QUE PROBABLEMENTE OCURRIRÁN EN LOS PÁRAMOS DE NOROESTE SUDAMERICA Y EL SUR DE CENTROAMERICA.

Supporting Information to the paper 'Páramo *Calamagrostis* s.l. (Poaceae): An updated list and key to the species known or likely to occur in páramos of NW South America and southern Central America including two new species, one new variety, and five new records for Colombia' Steven P. Sylvester, Robert J. Soreng, William J. Bravo-Pedraza, Lia E. Cuta-Alarcon, Diego Giraldo-Cañas, Jose Aguilar-Cano, Paul M. Peterson. Phytokeys (2019).

Este clave cubre las 54 especies de *Calamagrostis* s.l. aceptados que ocurren en páramos (ver listado de especies en el manuscrito principal). Las especies que no fueron incluidas en la clave están mencionadas en la sección 'excluded or ambiguous species' en el manuscrito principal.

1. Flosculo siempre estipitado (artejo inferior de la raquilla prolongado entre las glumas y el antecio) (primer flosculo estipitado), estipete de 0.4−4 mm long. (a veces un poco menor, a 0.2 mm, en *Deschampsia parodiana, D. santamartensis*, *Calamagrostis boyacensis*, y *C. ramonae* asi varias espiguillas deberían ser revisados), cilíndrico, dilatado hacia la articulación (se observa en la parte inferior de las glumas cuando se cae el antecio); hojas con o sin estipula lígular presente entre la vaina y la lamina 2
- Flosculo usualmente sésil (artejo inferior de la raquilla no, o no conspicuamente, prolongado entre las glumas y el antecio) (primer flosculo no conspicuamente estipitado), estipete menor de 0.3 mm long.; hojas sin estipula lígular presente entre la vaina y la lamina 21

2 (1). Antecio sin arista 3
- Antecio aristado, arista recta o retorcida y geniculada (cf. *Deschampsia aurea* a veces mucronada) 4

3 (2). Plantas 9−20 cm de alto, con hojas formando una mata basal hasta 12 cm de alto que es mucho menor que los culmos exertas; laminas cortas y anchas, 2.5−8 cm long., 1.5−2.5 mm de ancho cuando plegada, fuertemente plegadas; inflorescencia espiciforme, de 3−5.5 cm long., 1.5−2.5 cm de ancho; raquilla prolongación ausente o pequeño y glabrescente .... ***Deschampsia santamartensis***
- Plantas 26−110 cm de alto, con laminas basales y caulinares que forman macollas de medio tamaño con algunas laminas caulinares que sobrepasan la inflorescencia; laminas de las innovaciones y la parte inferior de los culmos fértiles 11−22 cm long., 0.35−1.2 mm de ancho cuando enrollada o plegada, angostas y conduplicadas o filiformes y involutas o convolutas, raramente completamente planas; laminas de la parte superior de los culmos fértiles 2.9−25 cm long., 2−7 mm de ancho cuando aplanada, planas, conduplicadas, o convolutas hacia sus ápices; inflorescencia abierta a semicontraida, 10−25 cm long., (3−)5−8 cm de ancho; raquilla prolongación presente y con pelos largos alcanzando desde ¾ hasta el ápice de la lema .... ***Deschampsia podophora* var. *mutica***

4 (2). Inflorescencia en forma de espiga densa, usualmente < 3 cm de ancho 5
- Inflorescencia abierta a semi-contraida, usualmente > 3 cm de ancho (*C. cleefii, C. guamanensis* y *C. pisinna* a veces a 1 cm de ancho, pero estas son generalmente laxas y pauciespiculadas) 13

5 (4). Pelos del callo cortas o ausentes, usualmente < 1 mm long., sin alcanzar la mitad del tamano de la lema; arista geniculada, retorcida, > 5 mm long. 6
- Pelos del callo largas, llegando desde la mitad del tamano de la lema hasta pasando su apice (a veces mas corta en *C. teretifolia* y *Deschampsia podophora*); arista recta, < 5 mm long. (geniculada y 5.5−8 mm long. en *C. boyacensis*)... 8

6 (5). Callo glabro; arista c. 5 mm long.; laminas foliares glabras, escabrosas; lema 3.7−5 mm long., bifida.. ***C. chaseae*** (en parte)
- Callo piloso, con pelos cortos usualmente < 1 mm long.; arista (5−)6−7(−8.8) mm long.; lamina foliar ciliada (*C. violacea*) o densamente piloso abaxialmente y adaxialmente (*C. mollis*); lema 3.8−6 mm long., su apice 4-dentado (*C. violacea*) o terminando en 4 setae delgadas (*C. mollis*)... 7

7 (6). Anteras de 0.4−0.6 mm long.; apice de la lema terminando en 4 setas delgadas; laminas foliares densamente pilosos abaxialmente y adaxialmente ....***C. mollis***
- Anteras 1.6−2.2(−2.4) mm long.; apice de la lema 4-dentado; laminas foliares glabras en la superficie pero usualmente ciliadas en el margen...***C. violacea***

8 (5). Arista 5.5−8 mm long., geniculada y retorcida, claramente sobrepasando las glumas; anteras 0.5−0.7 mm long.; espiguillas (5−)6−7.5 mm long.; raquilla con pelos largos alcanzando el apice de la lema o sobrepasándolo brevemente ....***C. boyacensis***
- Arista < 5.5 mm long., recta, sin alcanzar a poco pasando el apice de las glumas; anteras 0.5−2.6 mm long. (solo 0.5−0.6 mm long. en *D. ovata*, raramente 0.7 mm long. en *D. podophora*); espiguillas 3.5−14 mm long.; raquilla con pelos llegando desde 2/3 a sobrepasando la longitud de la lema 9

9 (8). Inflorescencias con tintes verdes o purpureas, a menudo interrumpidas, ovada a angostamente elliptica; espiguillas 3.5−6 mm long.; raquilla con pelos alcanzando desde 2/3 de la lema hasta casi el apice de la misma... 10
- Inflorescencias doradas y brillantes, continuas y sin interrupción, oblongas, ovoides, ellipsoides, subesfericas, cilíndricas, o capitadas; espiguillas (5−)7−14 mm long.; raquilla con pelos usualmente alcanzando o sobrepasando brevemente el apice de la lema, exceptionalmente mas cortas 11

10 (9). Inflorescencias usualmente interrumpidas, de contorno lobulada, ovada a elliptica, 10−25 cm long. × 3−8 cm de ancho; glumas 3.5−5.5 mm long.; lema 2.4−3.5 mm long.; callo con pelos llegando desde 1/3 hasta casi el apice de la lema; raquilla con pelos alcanzando desde 4/5 de la lema hasta casi el apice de la misma.........***Deschampsia podophora*** (en parte)
- Inflorescencias usualmente solo un poco interrumpidas, angostamente ellipticas, 5−6 cm long. × c. 1 cm de ancho; glumas c. 6 mm long.; lema c. 3.5 mm long.; callo con pelos llegando a c. ½ la longitud de la lema; raquilla con pelos alcanzando desde 2/3 a 4/5 de la longitud de la lema... ***C. teretifolia***

11 (9). Anteras 0.5−0.6 mm long.; glumas (6.2−)7.8−14 mm long.; lígula (estipula lígular) acuminada, 6−18 mm long. .... ***Deschampsia ovata***
- Anteras 1−2.6 mm long.; glumas 5−8 mm long.; estipula lígular acuminada (*D. chrysantha*) o lígula bifida en su apice (*D. aurea*), 0.7−20 mm long. 12

12 (11) Flosculo generalmente mide ½ o < ½ la longitud de las glumas; anteras 0.9−1.6 mm long.; callo con pelos llegando desde ½ a ¾ la longitud de la lema; arista insertada en el tercio superior de la lema y no sobrepasando el apice de la lema, o insertada en el tercio inferior de la lema y sobrepasando el apice de la lema, a veces ausente; estipula ligular inconspicua (exceptionalmente ausente en algunas hojas); lígula con apice bífida, 0.7−20 mm long.. ***Deschampsia aurea***
- Flosculo usualmente mayor que la ½ de la longitud de las glumas; anteras 1.6−2.6 mm long.; callo con pelos casi alcanzando o sobrepasando el apice de la lema; arista insertada en el tercio inferior de la lema y no alcanzando el apicede la lema o pasandolo brevemente; estipula ligular evidente, hialina, acuminada, con 2 quillas conspicuas que desvanecen hacia el apice, (0.7−)7−20 mm long.. ***Deschampsia chrysantha***

13 (4). Arista recta o levemente curvada, 1.5−4 mm long., usualmente no pasando los ápices de las glumas o solo pasandolos brevemente; espiguillas formando glomérulos mas o menos desarrollados en la parte distal de las ramas 14
- Arista geniculada y retorcida, > 5 mm long., facilmente sobrepasando los ápices de las glumas (a veces recta en *C. boyacensis* y *C. cleefii* pero luego >5 mm long.); espiguillas no glomeradas 16

14 (13). Anteras 0.4−0.5 mm long.; raquilla 1−1.2 mm long., poco pilosa con pelos que usualmente no alcanzan el apice de la palea; inflorescencias dorado-brillantes, laxas con ramas pendientes y las espiguillas glomeradas en la parte distal de las ramas; plantas usualmente altas, 50−80 cm de alto .... ***Deschampsia parodiana***
- Anteras (0.7−)1.2−2.5 mm long.; raquilla (0.8−)1−2.5 mm long., poco pilosa con pelos que usualmente no alcanzan el apice de la palea (*D. eminens*) o pilosa con pelos que alcanzan el ápice de la pálea o lema o lo pasen ampliamente (*D. podophora*); inflorescencias dorado-brillantes (*D. eminens*) o verdes con tintes purpureas (*D. podophora*), laxas con ramas pendientes y las espiguillas glomeradas en la parte distal de las ramas o subespiciformes y espiculadas desde la base; plantas altas o pequenas, 20−130 cm de alto.............................. 15

15 (14). Inflorescencias dorado-brillantes, laxas con ramas pendientes y las espiguillas dispuestas en glomérulos distales o proximales de 1−2 cm diam.; anteras 1.6−2.5 mm long.; raquilla (0.8−)1−1.8 mm long., poco pilosa, con pelos que usualmente no llegan al apice de la palea; plantas (36−)50−130 cm de alto .... ***Deschampsia eminens***
- Inflorescencias verdes con tintes purpureas, laxas con ramas pendientes y las espiguillas glomeradas en la parte distal de las ramas o subespiciformes y espiculadas desde la base; anteras (0.7−)1.2−1.5(−1.9) mm long.; raquilla (1.2−)1.4−2.5 mm long., pilosa, pelos que alcanzan el ápice de la pálea o lema o lo pasen ampliamente; plantas 20−75(−110) cm de alto
.... ***Deschampsia podophora*** (en parte)

16 (13). Lema bifurcada hasta casi la mitad, la arista saliendo de la base de la hendidura de la bifurcación 17
- Lema dentada, no fuertemente bifurcada; arista saliendo del dorso de la lema, no de entre una hendidura profunda de la lema 18

17 (16). Articulación entre la lámina y la vaina hinchada; laminas plegadas, 2−3 mm de ancho cuando desplegada; quillas de las glumas lisas; callo con pelos largos que alcanzan o sobrepasan la mitad de la lema; raquilla usualmente capitada, llegando a ¾ la longitud de la lema o más, los pelos largamente sobrepasando el ápice de la lema
.... ***C. guamanensis*** (en parte)

- Articulación entre la lámina y la vaina no hinchado; laminas planas o plegadas, 1−5.2 mm de ancho cuando desplegada; quillas de las glumas escabrosas; callo con pelos que no llegan a mas que 1/3 de la lema; raquilla no capitada, usualmente llegando a la mitad de la lema, los pelos usualmente solo alcanzando el apice de la palea (alcanzando o pasando el apice de la lema en especimines del este de Colombia) ....***C. pisinna*** (en parte)

18 (16). Pelos del callo y la raquilla retorcidas; anteras c. 0.5 mm long.; articulación entre la lámina y la vaina hinchado; arista retorcida (usualmente más que dos veces) y geniculada en dirección hacia la base de la lema, c. 5 mm long.; quilla de las glumas escabrosas; plantas 30−50 cm de alto, con laminas foliares largas y planas, 8−19 cm long., 3−4 mm de ancho, escabrosas adaxialmente y abaxialmente .... ***C. ramonae***
- Pelos del callo y la raquilla rectas; anteras 1−2.4 mm long.; articulación entre la lámina y la vaina no hinchado; arista retorcida y geniculada, brevemente retorcida en su base y curvada, o recta, mirando hacia la dirección opuesta de la base, (5−)5.4−8(−8.8) mm long.; quilla de las glumas lisas o escabrosas; plantas 10−30(−37) cm de alto, con laminas foliares mas cortas, 1.5−11(−14) cm long., angostas o anchas, convolutas, subinvolutas, conduplicadas o planas, por lo menos la superficie adaxial lisa 19

19 (18). Lema (3.8−)4.4−5(−5.8) mm long.; anteras 1.6−2.2(−2.4) mm long.; arista retorcida (usualmente más que dos veces) y geniculada, insertada 1−1.6 mm de la base de la lema; quilla de las glumas escabrosas o ciliadas; lígula (1.2−)1.6−3.2(−3.8) mm long. ....***C. violacea***
- Lema 2.2−2.8 mm long.; anteras 1−1.5 mm long.; arista brevemente retorcida en su base y geniculada a curvada o recta, insertada cerca a la base (*C. cleefii*) o en el tercio medio de la lema (*C. boyacensis*); quilla de las glumas lisas; lígula c. 0.5 mm long. (*C. cleefii*) o 5−17 mm long. (*C. boyacensis*).. 20

20 (19). Arista insertada cerca a la base de la lema, c. 5 mm long.; lígulas c. 0.5 mm long.; laminas foliares 2−5 cm long... ***C. cleefii***
- Arista insertada en el tercio medio del dorso de la lema, 6−7 mm long.; lígulas 5−17 mm long.; laminas foliares 5−11 cm long. ....***C. boyacensis***

21 (1). Prolongación de la raquilla ausente; callo del antecio con pelos sedosos que alcanzan las ½ a ¾ de la longitud de la lema; láminas planas; antecio con el callo redondeado, no recurvado; apice de la lema obtuso y brevemente hendido; glumas lanceoladas; panícula abierta ....***C. llanganatensis***
- Prolongación de la raquilla presente y saliendo de la base del antecio, bien desarrollada y pilosa a corta y glabra (a veces muy corto en e.g. *C. bogotensis*); combinación de las otras caracteres mencionadas arriba no presentes 22

22 (21). Callo con pelos que alcanzan desde la mitad a más de la longitud de la lema; panoja laxa y abierta, usualmente piramidal, a veces pauciespiculada 23
- Callo con pelos muy breves o que alcanzan hasta el 1/3 inferior de la lema; panoja laxa y abierta a densa y espiciforme.. 27

23 (22). Antecio con el callo recurvado; lema 3.5−4 mm long.; anteras 0.4−0.8 mm long.; láminas planas o raramente subconvolutadas; plantas 60−130 cm de alto 24
- Antecio con el callo redondeado o raramente agudo, no recurvado; lema 2−3 mm long.; anteras 0.8−1.5 mm long.; láminas planas, conduplicadas, convolutas o involutas; plantas de c. 60 cm (*C. steyermarkii*) o 6−25 cm de altura 25

24 (23). Pelos del callo poco menores que la longitud de la lema, excepcionalmente llegando al apice de la lema; las glumas superan brevemente la longitud del antecio, en 0.5−0.6 mm; lema con la arista fija en el tercio medio del dorso; lígula 1−4 mm long. ....***C. rupestris***
- Pelos del callo sobrepasando el apice de la lema; las glumas superan ampliamente al antecio en 1.5−6 mm long.; lema con arista dorsal fija en el tercio superior; lígula generalmente 1−1.5 mm long. ....***C. viridiflavescens***

25 (23). Plantas formando macollas de c. 65 cm de altura con láminas convolutas de 15−25 cm long.; inflorescencia c. 18 cm long.; lígula obtusa, 2−2.5 mm long.; callo con pelos densos que llegan desde ½ a ¾ la longitud de la lema; flosculo sésil, artejo inferior de la raquilla no prolongado entre las glumas y el antecio.......................................***C. steyermarkii***
- Plantas formando un césped basal corto de 6−25 cm de altura con láminas convolutas, conduplicadas o planas de 2−8 cm long.; lígula truncada u obtusa, < 1 mm long.; callo con pelos densos que llegan desde ½ la longitud de la lema a sobrepasar el apice de la lema; flosculo brevemente estipitado, artejo inferior de la raquilla prolongado entre las glumas y el antecio por 0.1−0.3(−1) mm 26

26 (25). Anteras 1.1(−1.5) mm long.; arista c. 5 mm long.; callo con pelos que alcanzan el apice de la lema; raquilla c. 2 mm long., con pelos que alcanzan el ápice de la lema..***C. cleefii***
- Anteras 0.8−1 mm long.; arista 7−9 mm long.; callo con pelos que alcanzan la mitad de la longitud de la lema; raquilla c. 1.5 mm long., con pelos largos que superan el apice de la lema ....***C. guamanensis*** (en parte)

27 (22). Panoja laxa y abierta, usualmente piramidal, generalmente mayor de 8 cm long., con ramificaciones verticiladas o semiverticilladas, divergentes, rectas o flexuosas, a veces las inferiores contraídas y péndulas, o con las ramificaciones inferiores distanciadas entre sí, mucho mayores que las superiores, a veces péndulas (*C. mandoniana*) 28
- Panoja subespiciforme, fusiforme, elíptica o capitada, ramificaciones laterales contraidas, pauciespiculadas o multiespiculadas, frecuentemente interrumpidas hacia la base 37

28 (27). Arista suprabasal, insertada a 0.3−0.6 mm de la base del dorso de la lema; lema 3−4 mm long. 29
- Arista basal o medial, insertada a 1−2.5 mm de la base del dorso de la lema; lema 3−7 mm long. 30

29 (28). Raquilla c. 0.9 mm long., casi glabra; lema con el ápice bífido; arista igual o más corta que la lema, no superando las glumas; lígula 1.5−2 mm long. .... ***C. divergens***
-Raquilla 1.5−2 mm long., poco pilosa con pelos largos y ralos; lema con el ápice dentado con nervios brevemente escurrentes; arista superando brevemente las glumas; lígula oblonga, > 4 mm long. .... ***C. naiguatensis***

30 (28). Raquilla glabra excepto por unos tricomas cortos en la base, usualmente no pasando la mitad de la lema; glumas 5−6 mm long.; lema c. 5 mm long.; ápice de la lema diminutamente bífida; arista c. 4.5 mm long. .... ***C. scaberula***
-Raquilla pilosa, los pelos cortos o largos pero en ambos casos llegando de ¾ a sobrepasar el apice de la lema; combinación de caracteres mencionadas arriba no presentes..................... 31

31 (30). Prolongación de la raquilla (sin tener en cuenta los pelos) llegando de ¾ hasta casi el ápice de la lema, generalmente con pelos < 1 mm long. que usualmente no alcanzan el ápice de la lema; arista (2−)4.5−7 mm long.; glumas 3−4(−5) mm long.; lema c. 3−3.9(−4.8) mm

long., apice de la lema truncado, irregularmente denticulado o bidentado; ramas y pedicelos usualmente lisas, raras veces escabriusculas; inflorescencia con ramas patentes; anteras 2−2.3 mm long. 32
- Prolongación de la raquilla (sin tener en cuenta los pelos) llegando a la mitad de la longitud de la lema (a veces a 2/3 en *C. macrophylla* y *C. planifolia*), con pelos largos, > 1 mm long., alcanzando el apice de la lema o pasándolo brevemente en *C. mandoniana* y *C. planifolia*, o llegando al apice de la palea en *C. macrophylla, C. pisinna* y *C. secunda* (*C. planifolia* a veces con pelos llegando solo a 2/3 pero solo tiene (1−)2 anteras); arista 5−9 mm long.; glumas 3−8.2 mm long.; lema 3.4−6.2 mm long.; apice de la lema hendido, 2-lobulado, o denticulado; ramas y pedicelos escabriusculos o escabrosas; inflorescencia con ramificaciones flexuosas, poco divergentes (péndulas en *C. mandoniana*); anteras 0.8−2.8 mm long. 33

32 (31). Láminas foliares fuertemente rizadas, crespas, formando un césped basal hasta 20 cm de alto en plantas maduras y mucho menor que la mitad de la longitud de las cañas floríferas; laminas foliares deciduas, desarticulándose fácilmente cuando maduras y cubriendo el piso alrededor de las plantas ....***C. crispifolius***
- Láminas foliares rectas, formando macollas 40−60(−107) cm de alto, hojas mayores que la mitad de la longitud de las cañas floríferas; laminas foliares no obviamente deciduas
.... ***C. effusa***

33 (31). Lema bifurcada hasta casi la mitad, la arista saliendo de la base de la hendidura de la bifurcación; anteras 2(−3), 0.9−1 mm long.; espiguillas 3.5−5(−6) mm long.
....***C. pisinna*** (en parte)
- Lema no fuertemente bifurcada, la arista saliendo del dorso de la lema, no de entre una hendidura profunda de la lema; anteras 2 (*C. planifolia*) o 3; espiguillas 3−8.2 mm long. 34

34 (33). Anteras 2, raramente 1, 1−1.4(−2) mm long.; glumas (3−)3.6−6 mm long.; lema 3.4−4.5(−5.5) mm long.; apice de la lema 2-lobulado, lóbulos bidentados; laminas basales generalmente planas, 1.8−7 mm de ancho .... ***C. planifolia*** (en parte)
- Anteras 3, 2−2.8 mm long.; glumas (5−)5.8−8.2 mm long.; lema (4.4−)5−6.2 mm long.; apice de la lema hendido y 4-dentado (*C. mandoniana*), bífido con dientes aristulados (*C. macrophylla*), o bífido y 4-dentado (*C. secunda*); laminas basales usualmente cilíndricas o subcilindricas, involutas o convolutas, a veces con los superiores abriendo a ser planas (*C. macrophylla, C. mandoniana*) 35

35 (34). Pelos de la raquilla usualmente llegando al apice de la lema o pasándolo brevemente; inflorescencia (24−)28−52 cm long., con ramas péndulas; ramas inferiores 10−16 cm long.; apice de la lema hendido y 4-dentado; lígula 2−12 mm long.; laminas planas o cilíndricas y involutas, de c. c. 3.3−3.6 mm ancho cuando desplegada............................... ***C. mandoniana***
- Pelos de la raquilla llegando de ½ a 4/5 de la longitud de la lema, usualmente no pasando la palea; inflorescencia 15−30 cm long.; ramas inferiores usualmente < 10 cm long.; apice de la lema bifido, a menudo con los dientes aristulados; lígula 3−6 mm (posiblemente mas larga?) long.; laminas cilíndricas o subcilindricas, involutas o convolutas, a veces con los superiores abriendo a ser planas 36

36 (35). Laminas superiores a menudo abriendo a ser planas, generalmente anchas, 2−4 mm ancho, involutas en la parte inferior; lígula con apice bífida y los segmentos alargados y lanceolados, lisa, 3−6 mm long. (posiblemente mas larga?); lema 5−7 mm long., apice bífida con dientes aristuladas...***C. macrophylla***

- Laminas generalmente cilíndricas o subcilindricas, involutas o convolutas, de c. 2 mm ancho cuando desplegada; lígula subtrigona, algo escabrosa, c. 4 mm long.; lema c. 4.7 mm long., apice bífida con dientes sin aristulas ....***C. secunda***

37 (27). Anteras 0.2−0.6 mm long., generalmente adheridas al ápice del fruto, flores generalmente cleistogamas (NB. *C. jamesonii* y *C. curta* usualmente son casmogamos pero anteras miden 0.4−0.6 mm long.); apice de la lema terminado en 4 arístulas o 4 dientes deltoides, aristados o erosos; estilopodio bien desarrollado, 0.4−0.6 mm long. (estilopodio ausente o breve en *C. curta* y *C. jamesonii*).. 38
- Anteras > 1 mm long., libres, flores casmógamas; apice de la lema hendido, 2−4-dentado, dientes regulares o irregulares, no aristados; estilopodio no diferenciado, si presente breve o incipiente 48

38 (37). Raquilla escasamente pilosa o glabra, si pilosa los pelitos cortos, poco visibles, llegando a la mitad de la lema 39
- Raquilla con pelos desarrollados y largos pudiendo alcanzar desde 2/3 hasta el ápice de la lema (*C. curta* y *C. sclerantha* tiene pelos cortos que alcanzan desde la mitad hasta las ¾ partes del antecio) 45

39 (38). Lema con arista dorsal recta o levemente curvada, inserta en el tercio medio del dorso, generalmente menor que las glumas; cañas floríferas rígidas, engrosadas, levemente curvadas, con inflorescencia generalmente subincluida en la última vaina; raquilla glabra o glabrescente ....***C. rigescens***
- Lema con arista dorsal retorcida y geniculada, inserta en el tercio inferior del dorso, mayor que las glumas; cañas floríferas gráciles, erectas, con inflorescencias generalmente exertas; raquilla escasamente pilosa, pelitos cortos, a veces solo en el ápice 40

40 (39). Láminas, por lo menos las de las cañas floríferas, planas, tiernas y glabras (raramente con márgenes ciliadas) 41
- Láminas todas convolutas o plegadas, junciformes, rígidas, curvadas o flexuosas 43

41 (40). Lemas (2.2−)3−3.5(−4.2) mm long., usualmente escabrosas en toda su superficie; hojas heteromorfas, las de la caña florífera usualmente planas, tiernas y glabras (a raramente con márgenes ciliadas), usualmente más anchas que las láminas de las innovaciones, que son usualmente convolutas, pilosas en ambas caras o solamente con el margen piloso ....***C. heterophylla***
- Lemas (4.5−)5−7 mm long., lisas por lo menos en su base (*C. brevipaleata* escabroso hacia su apice); todas las hojas planas, isomorfas (*C. hirta*) o heteromorfas, pero no diferenciados en ancho y tamaño solo en que las de las innovaciones son pilosas mientras que las de las cañas floríferas son glabras (*C. brevipaleata*).. 42

42 (41). Lemas lisas por todo su superficie; hojas isomorfas, las hojas de la caña y aquellas de las innovaciones semejantes, planas y pilosas, usualmente cortas, 1−11 cm long. ....***C. hirta***
- Lemas lisas en su base y escabroso hacia su apice; hojas heteromorfas, las hojas de la caña glabras y las del césped villosas, largas, 10−25 cm long. ....***C. brevipaleata***

43 (40). Plantas de 1−5 cm de alto, formando densos cojines; láminas foliares obtusas, menores de 1 cm de largo, curvadas al ras del suelo; inflorescencias exertas, formadas por 3−10 espiguillas .... ***C. minima***

- Plantas de (1.5−)4−50 cm de alto, formando matas laxas, no en densos cojines; láminas foliares conduplicadas, agudas o naviculares, rectas o curvadas, pero no a ras del suelo; inflorescencias exertas o subincluidas en las vainas, pauciespiculadas o multiespiculadas ... 44

44 (43). Laminas filiformes, 0.2−0.4 mm de ancho cuando abierto, involutas, curvadas o flexuosas, excepcionalmente rectas; raquilla 0.4−0.9(−1.2) mm long., poco pilosa, pelos escasos hasta 1.3 mm long. que usualmente no llegan a la ½ de la lema; lema (2.6−)3.1−5.8 mm long.. ***C. vicunarum***
- Laminas > 1 mm de ancho cuando desplegada, plegadas, usualmente rectas; raquilla (0.8−)1−2 mm long., con pelitos cortos usualmente 0.5−1 mm long. que apenas alcanzan la ½ de la lema; lema 4.5−5 mm long.. ***C. fibrovaginata***

45 (38). Glumas con sus quillas cilioladas .... ***C. jamesonii***
- Glumas con sus quillas escabriúsculas a escabrosas 46

46 (45). Ápice de la lema con 4 arístulas escabrosas de 1−2.1 mm de largo, que igualan o sobrepasan el ápice de las glumas, al menos a la gluma superior ....***C. setiflora***
- Ápice de la lema con 4 dientes deltoides, membranáceos, de 0.2−0.5(−0.7) mm de largo, no aristulados, menores que las glumas 47

47 (46). Flores cleistogamas; estilopodio presente, 0.5−0.6 mm long.; inflorescencias 1.7−5 cm long., con muchas espiguillas, subespiciformes; glumas 4.5−5.2 mm long, 1-nervias, excepcionalmente la gluma superior 3-nervia ....***C. scleranthа***
- Flores casmogamos; estilopodio ausente; inflorescencias hasta 2 cm long., pauciespiculadas, subglobosas; glumas 4.8−6.5 mm long., la inferior 1-nervia, la superior 3-nervia .... ***C. curta***

48 (37). Antecios sin arista ....***C. ecuadoriensis***
- Antecios aristados, arista dorsal a subapical 49

49 (48). Raquilla con pelos largos y densos, que generalmente igualan o superan el apice de la lema, exceptionalmente solo alcanzan el apice de la palea en *C. planifolia* y *C. rigida* (*C. planifolia* a veces con pelos llegando solo a 2/3 pero solo tiene (1−)2 anteras) 50
- Raquilla barbada con pelos cortos o glabra, si barbada los pelos generalmente alcanzan hasta las 4/5 de a longitud de la lema, pero no alcanzan el apice de la palea (*C. macrophylla* a veces tiene pelos que llegan al apice de la palea) 55

50 (49). Laminas generalmente aplanadas, convolutas al secarse, 2−7 mm de ancho; androceo formado por (1−)2 estambres; anteras 0.8−1.4(−2) mm long. .... ***C. planifolia*** (en parte)
- Laminas permanentemente involutas o fuertemente conduplicadas por todo su largo; androceo formado por 3 estambres; anteras (1.8−)2−3 mm long. 51

51 (50). Laminas recurvadas o ligeramente arqueadas, 1−5(−9) cm long.; hojas formando un césped basal menor que la mitad de la longitud de las cañas floríferas; plantas 5−35(−50) cm de alto, formando matas pequeñas o medianas, a veces formando cojines laxos o densos; inflorescencias generalmente < 5 cm long.; arista no superando la longitud de las glumas .... ***C. spicigera***
- Laminas rigidas, erectas, duras, punzantes, (6−)10−30 cm long.; hojas no formando un césped basal notable, usualmente formando un césped mayor que la mitad de la longitud de las cañas floríferas (*C. killipii* puede ser variable); plantas de (0.15−)0.3−1.3 m de altura,

generalmente formando macollas medianas o grandes; inflorescencias (5−)9−30 cm long. (5−8 cm en *C. killipii*); arista no superando a largamente exerta de las glumas 52

52 (51). Inflorescencias 5−8 cm long.; plantas pequeñas, < 30 cm de alto; lígula c. 0.8 mm long.. ***C. killipii***
- Inflorescencias (8−)9−20(−30) cm long.; plantas generalmente grandes, (15−)23−100 cm de alto; lígula (1−)1.5−15 mm long.. 53

53 (52). Inflorescencias subespiciformes, erectas, de contorno oval, con tintes dorados o bronceados, ramificaciones contraídas, densamente espiculadas desde la base, a veces lobuladas y subnutantes; laminas conduplicadas, de 1−1.4 mm diam. cuando plegadas; lígula coriácea a cartacea, con apice obtusa a aguda, 1−10 mm long............................... ***C. glacialis***
- Inflorescencias flexuosas, verdosas o violáceas, discontinuas, ramificaciones mas o menos contraídas, generalmente desnudas en la parte proximal; laminas convolutas o conduplicadas, de c. 1 mm diam. cuando plegadas; lígula membranácea, acuminada, generalmente 8−15 mm long.. 54

54 (53). Lema (4.2−)4.5−5.2(−5.4) mm long.; arista 4−5.8 mm long., alcanzando el apice de las glumas o sobrepasándolo brevemente; raquilla con pelos que alcanzan desde las ¾ de la longitud de la lema hasta el apice del mismo .... ***C. rigida***
- Lema (5−)5.4−6.2(−6.6) mm long.; arista 5.4−7.4 mm long., que sobrepasa ampliamente las glumas; raquilla con pelos que superan ampliamente el apice de la lema, exceptionalmente lo igualan .... ***C. intermedia***

55 (49). Arista 0.7−4.5(−5) mm long., incluida o escasamente pasando las glumas, delicada y recta a poco retorcida (geniculada y saliendo por entre las glumas en *C. scabriflora* y *C. chaseae*); raquilla y callo glabra o escasamente pilosa, los pelos hasta 0.8 mm long., no llegando a la mitad del antecio 56
- Arista 5.5−9 mm long., exerta de las glumas, retorcida y/o a veces geniculada; raquilla usualmente con los pelos alcanzando desde la ½ hasta las ¾ de a longitud del antecio o el apice de la palea (pelos a veces cortos en *C. involuta* y *C. fuscata* pero aristas >5.5 mm long.) 60

56 (55). Arista insertada en el tercio inferior de la lema; anteras 1 57
- Arista insertada en la mitad o encima de la mitad de la lema; anteras 1−2 (*C. carchiensis,* posiblemente *C. chaseae*) o 3 58

57 (56). Arista insertada a 0.3−0.7 mm de la base de la lema, (2.3−)3−4 mm long., recta a poco geniculada .... ***C. bogotensis***
- Arista insertada a c. 1 mm de la base de la lema, c. 1.7 mm long., geniculada y saliendo de los bordes de las glumas .... ***C. scabriflora***

58 (56). Arista 0.7−1 mm long.; anteras 1 (raramente 2 en material del este de Colombia) ....***C. carchiensis***
- Arista c. 3−5 mm long.; anteras 3 (posiblemente 1 en *C. chaseae*, aunque se necesita mas revision).. 59

59 (58). Callo escasamente piloso con pelos cortos de c. 0.1 mm long.; flosculo sésil; arista c. 3 mm long., insertada 3/5 de la base de la lema **..** ***C. fulgida***

- Callo glabro; flosculo estipitado, con el artejo inferior de la raquilla prolongado entre las glumas y el antecio de 0.2 mm long.; arista c. 5 mm long., insertada en el tercio medio .... ***C. chaseae*** (en parte)

60 (55). Laminas curvadas, recurvadas, flexuosas o rectas; plantas con hojas formando un césped basal generalmente menor que la mitad de la longitud de las cañas floríferas; matas pequeñas o medianas con cañas floríferas 11−32 cm de altura; inflorescencias generalmente 5−12 cm long.; raquilla escasamente pilosa, los pelos usualmente llegando a la mitad de la lema y no pasando el ápice de la palea.. 61
- Laminas rigidas, erectas, duras, punzantes; plantas con hojas formando un césped mayor que la mitad de la longitud de las cañas floríferas; matas densas, 10−180 cm de altura; inflorescencias 9−31 cm long.; raquilla con los pelos alcanzando desde la ½ hasta las ¾ de a longitud de la lema o el apice de la palea.. 62

61 (60). Arista insertada en el tercio inferior de la lema; raquilla 1.3−1.4 mm long., esparcidamente pilosa, pelos cortos a casi glabra; anteras 0.7−1 mm long.; lema 3−4.1 mm long.; vainas basales viejas fibrosas; lígula 0.5−1.5 mm long.; glumas 5−5.7 mm long. ....***C. involuta***
- Arista insertada en el tercio medio de la lema; raquilla (1.4−)1.6−2.5 mm long., poco pilosa, los pelos no alcanzan el ápice de la pálea; anteras c. 1.5 mm long; lema (3.8−)4−4.6 mm long.; vainas basales viejas no fibrosas; lígula 1.2−2(−2.5) mm long.; glumas 5.6−6.2 mm long...***C. fuscata***

62 (60). Laminas superiores abriendo a ser planas, generalmente anchas, 2−4 mm de ancho, involutas en la parte inferior; panicula ligeramente abierta ....***C. macrophylla***
- Laminas convolutas o conduplicadas por todo su largo, 0.3−2 mm de ancho cuando plegada; panicula densa (*C. recta*) o ligeramente abierta (*C. tarmensis*) 63

63 (62). Gluma superior 3-nervia, nervios laterales breves, alcanzando el tercio inferior de la misma; inflorescencia erecta, subespiciforme, ramificaciones laterales cortas y adpresas; laminas escabrosas, erectas, punzantes, rigidas; glumas 5.4−8(−8.5) mm long.............***C. recta***
- Gluma superior 3-nervia, nervios laterales superando la mitad de la longitud de la misma (menores en *C. tarmensis* var. *tarijensis*); inflorescencia con ramificaciones laterales algo flexuosas; laminas erectas y rigidas con la cara adaxial escabrosa o escabroso-pubescente, o laminas algo flexuosas con la cara adaxial escabruscula (*C. tarmensis* var. *tarijensis*); glumas (4.4−)4.8−6.2(−7) mm long.. ***C. tarmensis***
